# Semaphorin-1a prevents *Drosophila* olfactory projection neuron dendrites from mis-targeting into select antennal lobe regions

**DOI:** 10.1371/journal.pgen.1006751

**Published:** 2017-04-27

**Authors:** Hung-Chang Shen, Sao-Yu Chu, Tsai-Chi Hsu, Chun-Han Wang, I-Ya Lin, Hung-Hsiang Yu

**Affiliations:** 1 Institute of Cellular and Organismic Biology, Academia Sinica, Taipei, Taiwan; 2 Graduate Institute of Life Sciences, National Defense Medical Center, Taipei, Taiwan; New York University, UNITED STATES

## Abstract

Elucidating how appropriate neurite patterns are generated in neurons of the olfactory system is crucial for comprehending the construction of the olfactory map. In the *Drosophila* olfactory system, projection neurons (PNs), primarily derived from four neural stem cells (called neuroblasts), populate their cell bodies surrounding to and distribute their dendrites in distinct but overlapping patterns within the primary olfactory center of the brain, the antennal lobe (AL). However, it remains unclear whether the same molecular mechanisms are employed to generate the appropriate dendritic patterns in discrete AL glomeruli among PNs produced from different neuroblasts. Here, by examining a previously explored transmembrane protein Semaphorin-1a (Sema-1a) which was proposed to globally control initial PN dendritic targeting along the dorsolateral-to-ventromedial axis of the AL, we discover a new role for Sema-1a in preventing dendrites of both uni-glomerular and poly-glomerular PNs from aberrant invasion into select AL regions and, intriguingly, this *Sema-1a*-deficient dendritic mis-targeting phenotype seems to associate with the origins of PNs from which they are derived. Further, ectopic expression of *Sema-1a* resulted in PN dendritic mis-projection from a select AL region into *adjacent* glomeruli, strengthening the idea that Sema-1a plays an essential role in preventing abnormal dendritic accumulation in select AL regions. Taken together, these results demonstrate that Sema-1a repulsion keeps dendrites of different types of PNs away from each other, enabling the same types of PN dendrites to be sorted into destined AL glomeruli and permitting for functional assembly of olfactory circuitry.

## Author summary

In the *Drosophila* olfactory system, olfactory projection neurons (PNs) are derived from four neural stem cells (called neuroblasts) during the development. Intriguingly, these PNs generate complex dendritic patterns within the primary olfactory center of the brain, the antennal lobe (AL), to relay odorant information from olfactory sensory neurons in the periphery to neurons in higher olfactory centers. In this study, we investigate how various types of PNs use a repulsive transmembrane protein Semaphorin-1a (Sema-1a) to establish appropriate dendritic patterns within the AL. Previously, Sema-1a was proposed to globally control initial PN dendritic targeting along the dorsolateral-to-ventromedial axis of the AL. In contrast, we disclose an unknown role of Sema-1a, in which this neuronal protein acts to keep dendrites of various types of PNs produced from different neuroblasts away from select AL regions, thereby enabling the dendrites of the same types of PNs to sort correctly into destined glomeruli within the developing AL for assembly of the functional olfactory neural circuitry.

## Introduction

In the olfactory system, odorant inputs are detected by olfactory sensory neurons (OSNs) in the periphery and converged into individual glomeruli of the primary olfactory center, termed the antennal lobe (AL) in *Drosophila* and the olfactory bulb in mice, where projection neurons (PNs in *Drosophila* and mitral/tufted cells in mice) relay these inputs to other brain regions for decoding [1]. In *Drosophila*, most PNs are generated from four neural stem cells (called neuroblasts) and therefore can be assigned into four neural lineages: anterodorsal PNs (adPNs) in the ALad1 lineage, ventral PNs (vPNs) in the ALv1 lineage, lateroventral PNs (lvPNs) in the ALlv1 lineage, and lateral PNs (lPNs) from a lateral group of mixed PNs and local interneurons (LNs) in the ALl1 lineage [2, 3] (also see S1 Fig). Among these PNs, dendrites of most types of adPNs and lPNs innervate individual glomeruli (as uni-glomerular PNs) within the AL [4–9], whereas many types of vPNs and lvPNs establish poly-glomerular dendritic arborization patterns in the AL [10, 11]. Intriguingly, various types of adPNs, lPNs, vPNs and lvPNs distribute their dendrites in distinct but overlapping patterns within the AL [11]. Elucidating the molecular mechanisms underlying how types of PNs within different neural lineages generate the complex patterns of dendritic arborizations in discrete AL glomeruli is crucial for comprehending the formation of the functional olfactory circuitry.

Since it is unclear whether the same molecular mechanisms are utilized to generate the complex dendritic patterns of adPNs, lPNs, vPNs and lvPNs, it is important to examine the roles of the same organizing cues in the formation of appropriate dendritic patterns for different types of PNs. For example, it has been previously reported that initial PN dendritic targeting in the developing AL is mediated through opposing gradients of repulsive semaphorin cues, Sema-2a/-2b, and a receptor for these cues, the transmembrane protein semaphorin-1a (Sema-1a). The ventromedial (VM) expression of secreted Sema-2a/-2b from degenerating larval OSN axons is proposed to influence PN dendritic elaboration that is dependent upon dorsolateral (DL) expression of membrane-tethered Sema-1a in PNs [12, 13]. Dendrites of DL1 adPNs and DA1 lPNs underwent a DL-to-VM shift when the whole animal was deficient for *Sema-2a/-2b*, or when *Sema-1a* was selectively removed from PNs, suggesting a crucial role for a repulsive Sema-2a/-2b gradient that is read by the receptor Sema-1a in setting up appropriate dorsal dendritic patterns for adPNs and lPNs [12, 13]. In contrast, RNAi knock-down of *Sema-1a* caused the dendrites of DA1 vPNs to no longer be constrained within the DA1 glomerulus, with a substantial fraction of these dendrites invading the DA3 glomerulus [14], implicating Sema-1a as a regulator of vPN dendritic morphogenesis. However, it is rather puzzling why the shifted DA1 vPN dendrites in the absence of Sema-1a, which are perpendicular to those of *Sema-1a*-deficient DL1 adPNs, do not exhibit a DL-to-VM shift, a prediction of the current model [12, 13]. Therefore, it is possible that the Sema-1a signal is transmitted differently in adPNs and lPNs compared to vPNs, or even that an alternative model accounts for these *Sema-1a* loss-of-function (LOF) dendritic phenotypes.

Here, using genetic LOF and rescue studies we identify a previously unknown role for Sema-1a in preventing aberrant dendritic invasion of both uni-glomerular and poly-glomerular PNs into select AL regions, including the DA3 glomerulus and the region close around the VC1 glomerulus; this role is distinct from previously explored functions of Sema-1a in global control of initial PN dendritic targeting along the DL-to-VM axis of the AL [12]. Intriguingly, the prevention of dendritic mis-targeting to the DA3 glomerulus mediated by Sema-1a seems to be PN-origin dependent, i.e., the occurrence of the *Sema-1a*-deficient dendritic mis-targeting phenotype only in the types of PNs derived from adPN and vPN neuroblasts but not from the lPN neuroblast. Further, ectopic expression of *Sema-1a* caused DA3 adPNs that normally send their dendrites to the DA3 glomerulus to mis-project their dendrites into adjacent glomeruli. Taken together, our results suggest that repulsive Sema-1a signals in adPNs, lPNs and vPNs keep different types of PN dendrites away from each other, ensuring that they instead navigate to their destined glomeruli to establish appropriate dendritic patterns for assembling the functional olfactory circuitry to decode odorant information from the external world.

## Results

### Loss-of-function of *Sema-1a* results in mis-targeting of vPN dendrites to the DA3 glomerulus

We sought to label adPNs (or lPNs) and vPNs in distinct colors that permits the simultaneous visualization of how the dendrites of different PN populations distribute within the AL during development. Applying the twin-spot MARCM (mosaic analysis with repressible cell markers) system [15], we induced independent fluorescent labeling of adPN (or lPN) and vPN neuroblasts of newly hatched larvae (NHL) to visualize larval-born-adPNs (or -lPNs) and -vPNs in two different colors (labeled by GAL4-GH146 and GAL4-MZ699) [6, 10]. We found that dendrites of adPNs (or lPNs) and vPNs were initially segregated at the early pupal stage, became apparently mixed at 48 hours APF and turned into fully intermingled in the adult AL (S2 Fig). The observation of dendritic mixing among adPNs (or lPNs) and vPNs in the developing AL raises an interesting question as to whether or not vPNs employ similar or different molecular mechanisms from those used by adPNs and lPNs to generate appropriate dendritic patterns during their morphogenesis.

**Fig 1:**
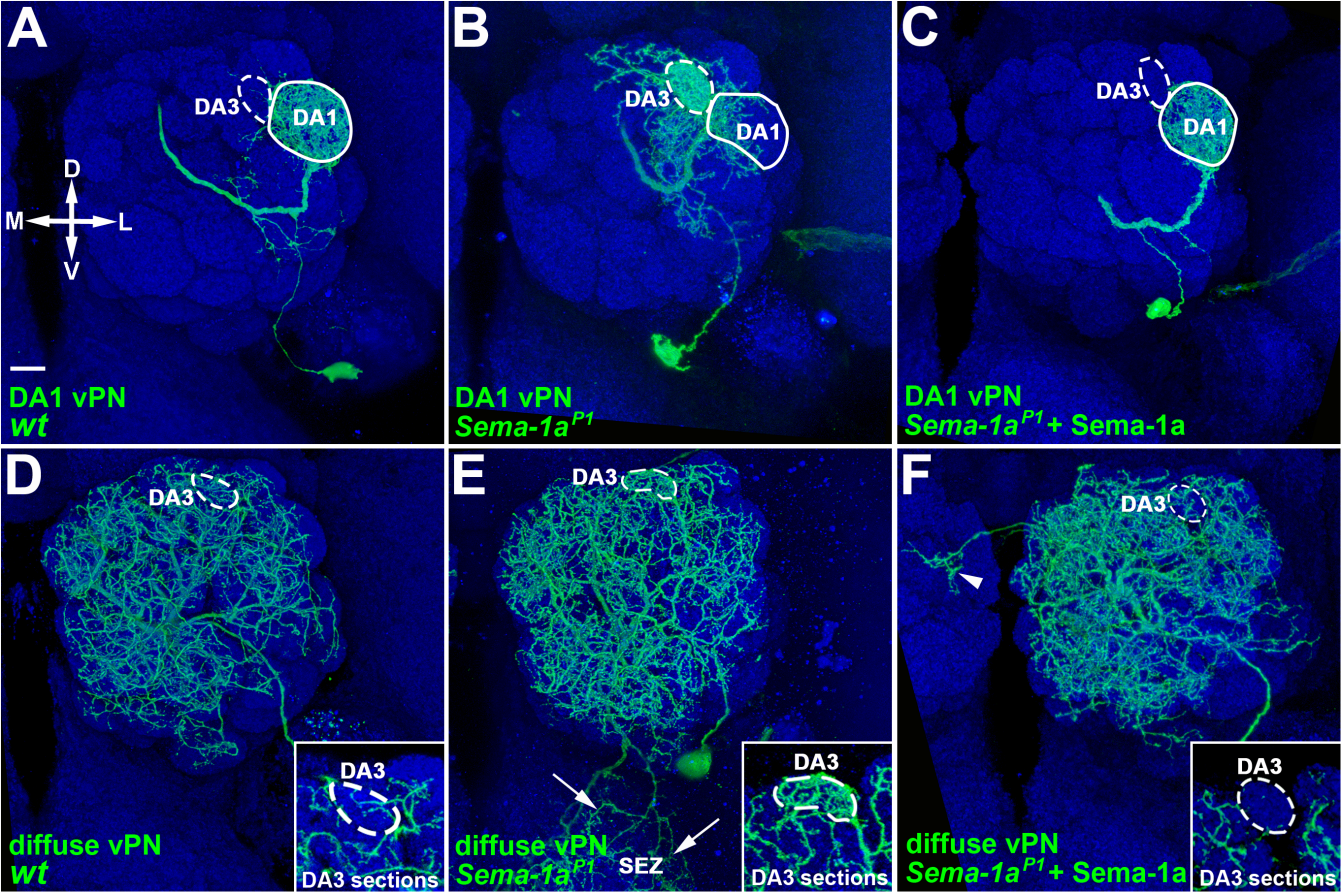
Dendrites of DA1 and diffuse vPNs mis-targeted to the DA3 glomerulus in the *Sema-1a*^*P1*^ mutant. Confocal images of DA1 (A-C) and diffuse (D-F) vPNs (green; labeled by GAL4-GH146) were flattened from sections covering the depth of the whole AL (A-F) and just the DA3 glomerulus (insets of panels D-F). (A, B, D and E) The DA3-glomerular dendritic mis-targeting phenotypes were observed in the *Sema-1a*^*P1*^ DA1 and diffuse vPNs (B and E), whereas no phenotype was observed in the wild-type samples (A and D). The DA3-glomerular dendritic accumulation in the *Sema-1a*^*P1*^ diffuse vPN was better appreciated in the confocal image flattened for the sections covering the depth of the DA3 glomerulus only (compare the green signal within dashed-circles in insets of panels D and E). A phenotype of aberrant neurite projection to the subesophageal zone (SEZ) was also observed in diffuse vPNs in the *Sema-1a*^*P1*^ mutant (arrows in E). (C and F) Both SEZ and DA3-glomerular dendritic mis-targeting phenotypes were rescued in the DA1 and diffuse vPNs by over-expression of Sema-1a in the *Sema-1a*^*P1*^ mutant. Intriguingly, dendrites hardly innervated the DA3 glomerulus in the *Sema-1a*^*P1*^ diffuse vPN with *Sema-1a* over-expression (compare the green signal within dashed-circles in insets of panels E and F). Aberrant midline-crossing dendrites were occasionally seen (arrowhead in panel F). The genotypes and orientation of the samples in all figures and supporting figures are summarized in S6 Table and arranged as medial (M) to the left, lateral (L) to the right, dorsal (D) to the up and ventral (V) to the bottom. Brain neuropiles (shown in blue) were stained with antibody against Bruchpilot (Brp) and the boundaries of the DA3 and DA1 glomeruli were marked with dashed-circles and circles in panels A-F and panels A-C, respectively. Scale bar: 10 μm in all panels and 15 μm in insets of panels C-F.

Previous work demonstrates that dendrites of DL1 adPNs and DA1 lPNs, as opposed to those of DA1 vPNs, have qualitatively distinct phenotypes in LOF studies of *Sema-1a* (see S3A Fig for the illustrative drawing of *Sema-1a*-deficient dendritic phenotypes observed in DL1 adPNs and DA1 vPNs) [12, 14]. To verify that mis-targeting of dendrites to the DA3 glomerulus we observed in the *Sema-1a* RNAi knock-down DA1 vPN (also see S3B–S3G Fig) actually resulted from the absence of *Sema-1a* rather than from an off-target effect of *Sema-1a* RNAi [16], we conducted MARCM experiments on a severe *Sema-1a* LOF mutation (*Sema-1a*^*P1*^) using GAL4-GH146, which labels four types of vPNs: DA1, diffuse, VA1lm and VL1 (Fig 1) [4, 5, 17]. In contrast to the wild-type DA1 vPN dendrites, which predominantly innervated the DA1 glomerulus (Fig 1A; Table 1), the *Sema-1a*^*P1*^ mutant DA1 vPN dendrites robustly mis-target into the DA3 glomerulus (Fig 1B; 95%, n = 21; Table 1). Notably, this DA3-glomerular dendritic mis-targeting defect was completely rescued by restoring the expression of Sema-1a in DA1 vPNs such that dendritic innervation was almost exclusively within the DA1 glomerulus (Fig 1C; Table 1). These results show that DA1 vPN dendrites aberrantly invade into the DA3 glomerulus when the expression of *Sema-1a* is disrupted.

**Table 1:** Phenotypic description of PNs in *wild-type*, *Sema-1a LOF* and rescue experiments in Figs 1, 2, 3 and 4 and S5 Fig.

*Sema-1a*-deficient PNs with a phenotype related to DA3-glomerular dendritic mis-targeting	type of PN (emb/larval)^a^	conditions of Sema-1a expression	total number (n)	% of DA3- glomerular dendritic mis-targeting	% of dendritic occupancy in Brp negative regions and/or partial glomerulus	% of other dendritic mis-targeting	notes
	DA4l adPN (emb; Fig 2)	*wt*	2	0%	0%	0%	
		*Sema-1a*^*P1*^	3	67%^b^	0%	0%	^b^ dendritic innervation in the ventrolateral part of the DA3 glomerulus
	DA4m adPN^e^ (emb; Fig 2 & S5)	*wt*	4	0%	0%	25%^d^	^d^ a few dendritic arbors around the DC1 glomerulus
		*Sema-1a*^*RNAi*^	9	78%^b^	0%	0%	^b^ partial dendritic innervation in the DA3 glomerulus
		*Sema-1a*^*P1*^	4	50%^b^	0%	100%^d^	^b^ partial dendritic innervation in the DA3 glomerulus ^d^ dendritic innervation in the SEZ
	DC1 adPN^e^ (emb; Fig 2)	*wt*	5	0%	0%	20%^d^	^d^ some dendritic arbors around the D glomerulus
		*Sema-1a*^*P1*^	3	67%^b^	0%	100%^d^	^b^ dendritic innervation in the ventrolateral part of the DA3 glomerulus ^d^ dendritic innervation to the region dorsal to the VC1 glomerulus
	DL4 adPN (emb; Fig 2)	*wt*	12	0%	0%	25%^d^	^d^ dendritic arbors in the DC1 glomerulus (17%) or ventral to the DP1m glomerulus (8%)
		*Sema-1a*^*P1*^	17	12%^b^	0%	18%^d^	^b^ substantial dendritic innervation in the DA3 glomerulus ^d^ dendritic arbors in the DC1 glomerulus (12%) or ventral to the DP1m glomerulus (6%)
	D adPN (larval; Fig 2)	*wt*	5	0%	0%	0%	
		*Sema-1a*^*P1*^	14	36%^b^	0%	0%	^b^ dendritic innervation largely in the dorsal part of the DA3 glomerulus
		*Sema-1a*^*P1*^+Sema-1a	13	0%	100%^c^	0%	^c^ dendritic innervation largely outside of the D glomerulus (Brp negative region)
	DA3 adPN (larval; Fig 3)	*wt*	5	0%	0%	0%	
		*Sema-1a*^*P1*^	23	0%	0%	4%^d^	^d^ dendritic innervation in the DA4l glomerulus (no DA3 glomerulus was found in the AL)
		*Sema-1a*^*P1*^+Sema-1a	16	0%	0%	100%^d^	^d^ dendritic innervation in the DL3 (63%), DA4l (12%) and DL3 plus DA4l (25%) glomeruli
	DC3 adPN (larval; Fig 2)	*wt*	10	0%	0%	0%	
		*Sema-1a*^*P1*^	9	100%^b^	0%	0%	^b^ full dendritic innervation in the DA3 glomerulus
		*Sema-1a*^*P1*^+Sema-1a	23	0%	0%	0%	
	VA1d adPN (larval; Fig 2)	*wt*	14	0%	0%	0%	
		*Sema-1a*^*P1*^	10	40%^b^	0%	0%	^b^ dendritic innervation in the ventral part of the DA3 glomerulus
		*Sema-1a*^*P1*^+Sema-1a	14	0%	100%^c^	0%	^c^ dendritic innervation in the ventral part of the VA1d glomerulus
	DA1 vPN (larval; Fig 1)	*wt*	17	0%	0%	0%	
		*Sema-1a*^*P1*^	21	95%^b^	0%	5%^d^	^b^ full dendritic innervation in the DA3 glomerulus ^d^ dendritic innervation in the DA4m glomerulus
		*Sema-1a*^*P1*^+Sema-1a	19	0%	0%	0%	
	diffuse vPN^e^ (larval; Fig 1)	*wt*	6	0%	0%	0%	
		*Sema-1a*^*P1*^	4	100%^b^	0%	100%^d^	^b^ heavy dendritic accumulation in the DA3 glomerulus ^d^ dendritic innervation in the SEZ
		*Sema-1a*^*P1*^*+Sema-1a*	7	0%	100%^c^	29%^d^	^c^ no/rare dendritic innervation in the DA3 glomerulus ^d^ dendritic innervation across midline to the contralateral AL
	DA3/VC1 adPN^e^ (emb; Fig 4)	*Sema-1a*^*P1*^	2	100%^b^	0%	100%^d^	^b^ heavy dendritic accumulation in the DA3 glomerulus ^d^ dendritic innervation to the region around the VC1 glomerulus

^**a**^ emb:embryonic-born; larval: larval-born.

^**b, c, d**^ see the description in the column of notes.

^**e**^ same types of PNs can be also seen in different classes of dendritic mis-targeting phenotypes in Table 2 and S5 Table.

It is unclear whether this dendritic mis-targeting phenotype is unique to DA1 vPNs or occurs in other types of vPNs (e.g., diffuse-, VA1lm- and VL1-vPNs) as well in the absence of *Sema-1a*. Interestingly, diffuse vPNs also exhibited a similar dendritic mis-targeting to the DA3 glomerulus in the *Sema-1a*^*P1*^ mutant: they accumulated in the DA3 glomerulus and aberrantly projected to the subesophageal zone (SEZ), in contrast to wild type, where the dendrites of diffuse vPNs were loosely distributed to nearly all of the AL glomeruli (Fig 1D–1E and their insets; 100%; Table 1). Both the DA3-glomerular dendritic accumulation and SEZ mis-projection phenotypes disappeared when *Sema-1a* was over-expressed in *Sema-1a*^*P1*^ diffuse vPNs (Fig 1F and its inset; Table 1). On the other hand, we did not observe the DA3-glomerular dendritic mis-targeting phenotype in the other two types of GAL4-GH146-positive vPNs, VA1lm- and VL1-vPNs, when the expression of *Sema-1a* was altered (S4 Fig; S1 Table). Taken together, the DA3-glomerular dendritic mis-targeting defect observed in the *Sema-1a*-deficient DA1- and diffuse-vPNs demonstrates that Sema-1a plays a crucial role in establishing appropriate dendritic patterns of both uni-glomerular and poly-glomerular vPNs, supporting a model that Sema-1a counteracts putative attractive force of the DA3 glomerulus, and this specific dendritic mis-targeting defect seems deviated from the prediction of the current model in which PN dendrites shift along the DL-to-VM axis of the AL in the absence of *Sema-1a* [12].

### adPN, but not lPN, dendrites that project near the DA3 glomerulus also exhibit DA3 dendritic glomerular mis-targeting defects in the *Sema-1a* loss-of-function mutant

**Fig 2:**
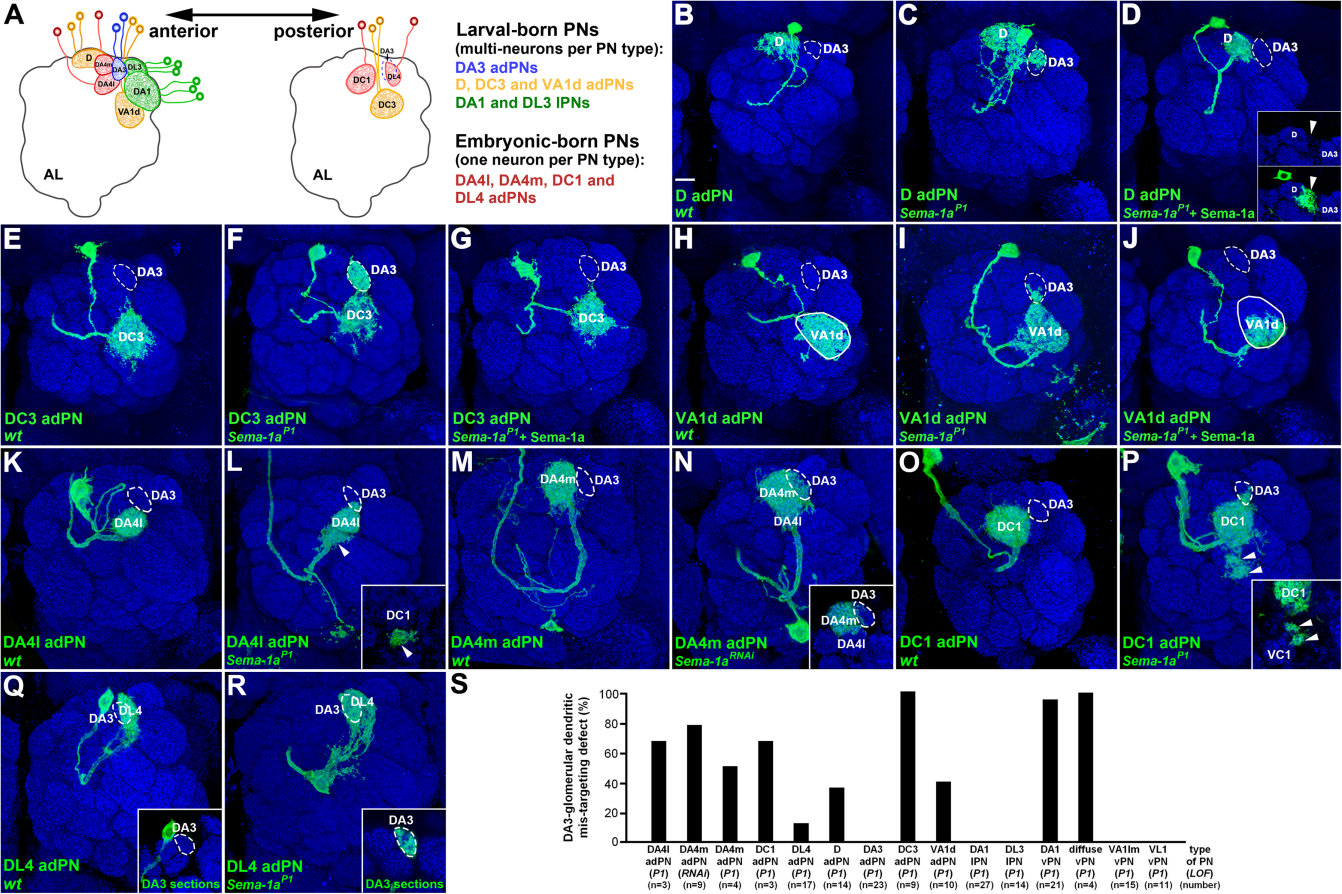
Dendrites of embryonic- and larval-born adPNs aberrantly invaded into the DA3 glomerulus in the absence of *Sema-1a*. (A) Relative positions of the AL glomeruli surrounding to the DA3 glomerulus (solid blue circled-line) and types of PNs with the dendritic projection to their destined glomeruli are illustrated in anterior and posterior sections of the schematic drawing. (B-R) Confocal images of D, DA4l, DA4m, DC1, DC3, DL4 and VA1d adPNs (green; labeled by GAL4-GH146 except for DA4m adPNs, which were labeled by R38B04-GAL4) were used to reveal their dendritic morphologies in the AL. DA3-glomerular dendritic mis-targeting phenotypes were observed in those adPNs when *Sema-1a* was deficient (C, F, I, L, N, P and R), whereas no dendritic phenotype was observed the wild-type adPNs (B, E, H, K, M, O and Q). Dendrites of the *Sema-1a*^*P1*^ DC1 adPN were also observed to mis-target the region close to the VC1 glomerulus (arrowheads in panel P and its inset). (D, G and J) The DA3-glomerular dendritic mis-targeting phenotypes were rescued in the *Sema-1a*^*P1*^ mutant by restoring the expression of *Sema-1a* in the DC3, D and VA1d adPNs. Dendrites of D- and VA1d-adPNs occupied the edge and outside of the D glomerulus and at the ventral portion of the VA1d glomerulus in the rescue experiments (arrowheads in insets of panel D and the green signal within circles of panels H and J). (S) Percentage of the DA3-glomerular dendritic mis-targeting phenotype in various types of adPNs, lPNs and vPNs was presented in the bar graph. Brain neuropiles (shown in blue) were stained with the antibody against Brp and the boundaries of the DA3 and VA1d glomeruli were marked with dashed-circles and circles in all panels and in panels H and J, respectively. Insets within panels were derived from single sections (D, L, N and P) or sections covering the depth of the DA3 glomerulus (Q and R) of the corresponding confocal images to highlight points of interest. Scale bar: 10 μm.

Since dendrites of wild-type DA1- and diffuse-vPNs are distributed close to the DA3 glomerulus, and since dendrites of *Sema-1a*-deficient DA1- and diffuse-vPNs mis-target into the DA3 glomerulus (Fig 1 and S3 Fig), we wondered whether Sema-1a signaling serves to prevent aberrant dendritic invasion into the DA3 glomerulus by surrounding PNs. To test this hypothesis, we examined *Sema-1a* LOF effects on the adPNs and lPNs which normally project their dendrites to surround the DA3 glomerulus (Fig 2): these adPNs and lPNs include DL3- and DA1-lPNs and VA1d-, DA4l-, DA4m- and D-adPNs, which project their dendrites clockwise to surround the DA3 glomerulus; they also include DL4-, DC3- and DC1-adPNs, which send their dendrites posteriorly covering the DA3 glomerulus (Fig 2A). Notably, all the PNs we examined except DA1- and DL3-lPNs mis-targeted their dendrites into the DA3 glomerulus in the absence of *Sema-1a* (Fig 2B–2S; S5 and S6 Figs; Table 1), similar to the dendritic mis-targeting phenotype we observed in the *Sema-1a*^*P1*^ DA1- and diffuse-vPNs (Fig 1). Among the adPNs we examined, DC3 adPNs displayed the most severe defect, with full dendritic invasion into the DA3 glomerulus in all *Sema-1a-*deficient animals (Fig 2E, 2F and 2S; Table 1). The rest of the adPN types exhibited differing degrees of penetrance and expressivity of the DA3-glomerular dendritic mis-targeting phenotype in the *Sema-1a*^*P1*^ mutant and *Sema-1a* RNAi knock-down samples (Fig 2B, 2C, 2H, 2I and 2K–2S; S5 Fig; Table 1). The DA3-glomerular dendritic mis-targeting phenotype in the *Sema-1a*^*P1*^ D-, DC3- and VA1d-adPNs was no longer observed when wild-type *Sema-1a* was over-expressed in these same PNs (Fig 2D, 2G and 2J; Table 1). Notably, in the rescue experiments the dendrites of D- and VA1d-adPNs remained situated on the edge and outside of the D glomerulus (insets of Fig 2D; 100%, n = 13; Table 1) and at the ventral portion of the VA1d glomerulus (Fig 2J; 100%, n = 14; Table 1), implicating that dendrites of the rescued and remaining wild-type adPNs may repel with each other. Taken together, the dendritic mis-targeting defect we observed in *Sema-1a*-deficient adPNs and vPNs suggests that the DA3 glomerulus serves as a select AL region for extending dendrites in the absence of *Sema-1a*. Intriguingly, in this DA3-glomerular dendritic mis-targeting phenotype, the PNs with dendrites that surround the DA3 glomerulus, including seven types of adPNs (D, DA4l, DA4m, DC1, DC3, DL4 and VA1d) and two types of vPNs (DA1 and diffuse), but not DA1- and DL3-lPNs, tend to aberrantly extend their dendrites into the DA3 glomerulus when *Sema-1a* is absent.

### Ectopic expression of Sema-1a in *Sema-1a* mutant DA3 adPNs causes dendritic mis-projection into the DL3 and DA4l glomeruli

**Fig 3:**
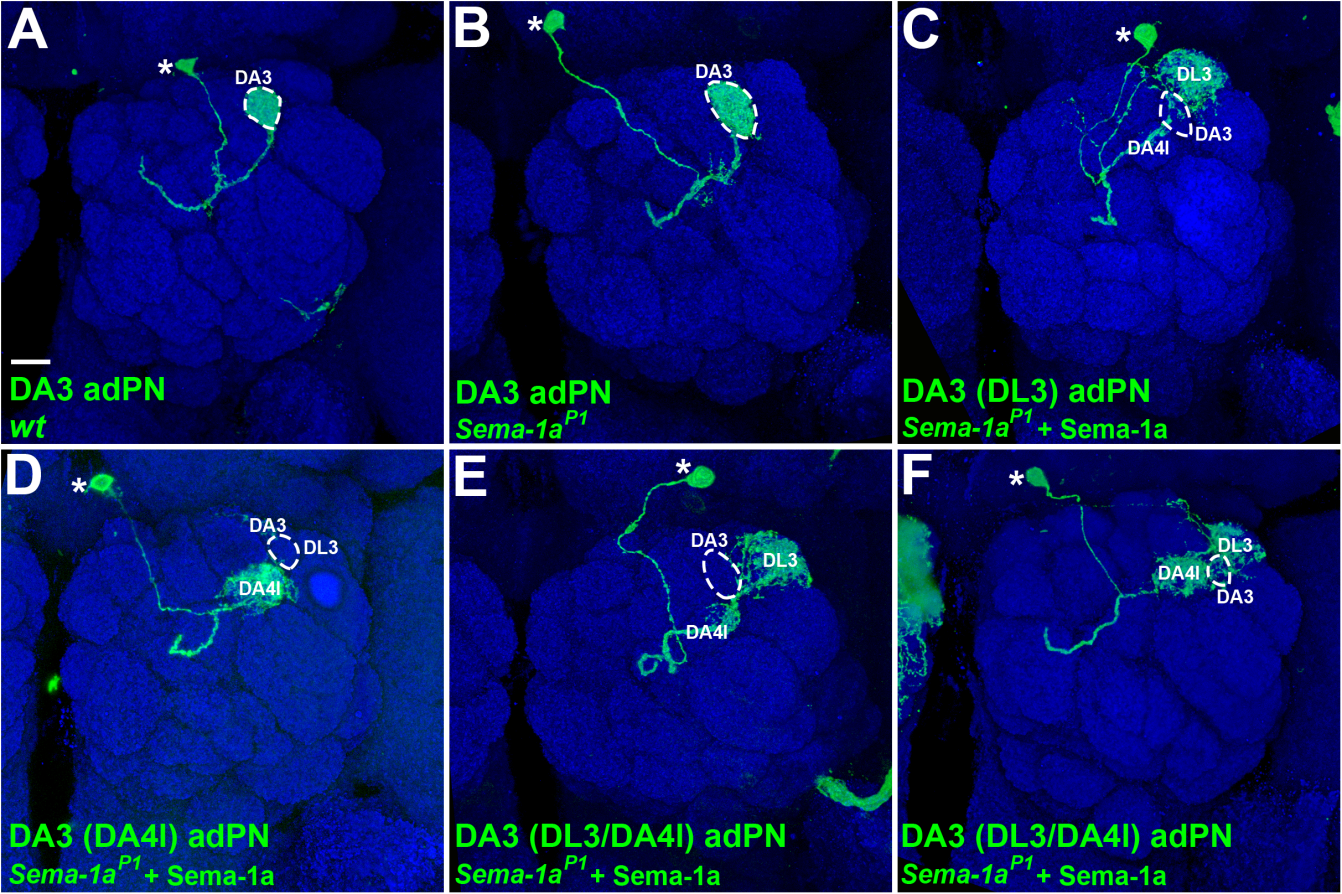
Manipulating *Sema-1a* expression resulted in DA3 adPN dendrites to mis-project away from the DA3 glomerulus to the DL3 and DA4l glomeruli. Confocal images of DA3 adPNs (green; labeled by GAL4-GH146) were used to reveal their dendritic patterns in the AL. (A and B) The dendritic phenotype was hardly found in the wild-type and *Sema-1a*^*P1*^ DA3 adPNs. (C-F) When *Sema-1a* was over-expressed in the *Sema-1a*^*P1*^ mutant, putative DA3 adPNs (judged by their birth-order) mis-projected their dendrites to the DL3 glomerulus in the majority of samples (C) and to the DA4l glomerulus (D) or both DL3 and DA4l glomeruli (E and F) in a few cases. Partial dendritic innervation into the lateral DA4l glomerulus was observed in panel E. Brain neuropiles (shown in blue) were stained with antibody against Brp and the boundary of the DA3 glomerulus was marked with dashed-circles in all panels. Soma positions of DA3 adPNs are indicated by asterisks in all panels. Scale bar: 10 μm.

The disappearance of dendrites from the DA3 glomerulus in diffuse vPNs observed in the *Sema-1a*^*P1*^ mutant with *Sema-1a* over-expression (Fig 1F and its inset) prompted us to ask how PN dendrites that normally project into the DA3 glomerulus (e.g., DA3 adPNs) would behave when *Sema-1a* expression is altered (Fig 3). Since dendrites of wild-type DA3 adPNs already distribute themselves into the DA3 glomerulus that attracts *Sema-1a*-deficient dendrites (Fig 2), we predicted that DA3 adPN dendritic projections to the DA3 glomerulus should remain unaffected when *Sema-1a* is mutated. Indeed, we found that DA3 adPNs rarely displayed abnormal dendritic phenotypes in the *Sema-1a*^*P1*^ mutant (Figs 2S, 3A and 3B; Table 1; see S2 Table for information on the birth-order of *Sema-1a*^*P1*^ DL1-, DA3- and DC2-adPNs in our synchronized MARCM experiments), implicating that the endogenous Sema-1a expression may be low (if there is any expression) and may not play a crucial role in the dendritic targeting of the DA3 adPNs. Furthermore, if Sema-1a counteracts the attraction in the DA3 glomerulus, DA3 adPN dendrites should be sensitive to an excessive level and ectopic time window of the Sema-1a gain-of-function paradigm. Since we did not systematically conduct the synchronized MARCM experiments to over-express *Sema-1a* in the wild-type PNs, we, instead, altered *Sema-1a* expression in DA3 adPNs by over-expressing *Sema-1a* in the *Sema-1a*^*P1*^ mutant PNs. Using this approach, we did not find any adPNs with dendritic projections into the DA3 glomerulus. Instead, we found many adPNs whose dendrites projected into the DL3 glomerulus in the majority of the cases (63%, n = 16; Table 1; Fig 3C) and in a few cases into the DA4l glomerulus (12%, n = 16; Table 1; Fig 3D) or both the DA4l and DL3 glomeruli (25%, n = 16; Table 1; Fig 3E and 3F) during the developmental time window for the generation of DA3 adPNs. We noted that our determination of the identity of these DL3/DA4l PNs as DA3 adPNs was based on their anterodorsal soma position, ruling out their being lPNs (Fig 3C–3F; the only wild-type DL3 PNs labeled by GAL4-GH146 are DL3 lPNs [6]). Further, the birth of these DL3/DA4l PNs occurred prior to the birth of DC2 adPNs but after the birth of DL1 adPNs in our synchronized MARCM experiments, establishing their identity as DA3 adPNs (S3 Table; the birth order of the embryonic-born DA4l adPN and larval-born DL1-, DA3-and DC2-adPNs has been reported previously [6]). Taken together, these results using manipulation of *Sema-1a* expression in DA3 adPNs reinforces our hypothesis that the DA3 glomerulus acts as a select AL region to attract nearby PN dendrites when counteracting Sema-1a signaling is absent.

### An additional PN dendritic-mis-targeting region in the AL is identified in the absence of *Sema-1a*

**Fig 4:**
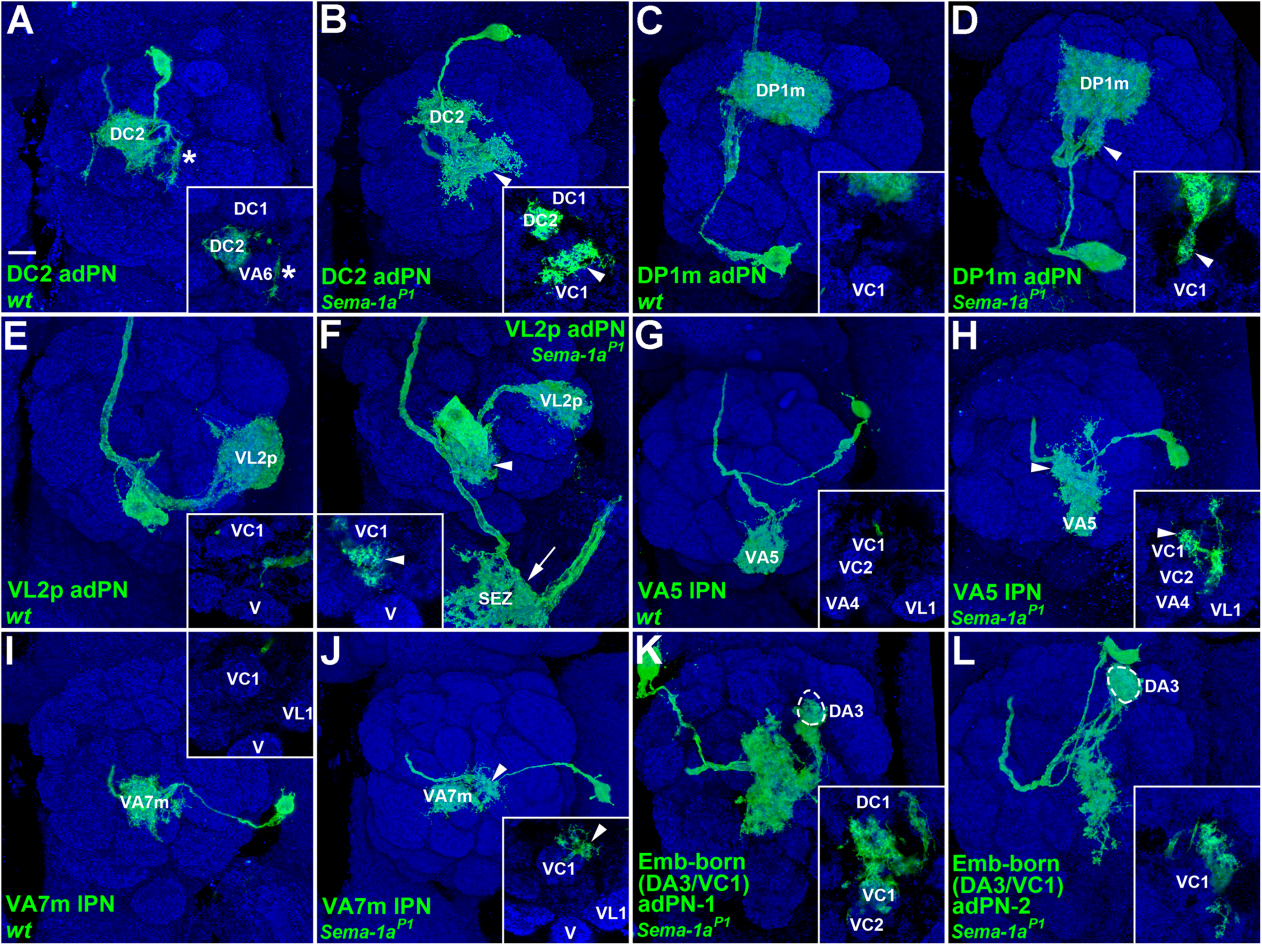
An additional select AL region around the VC1 glomerulus attracted PN dendrites in the absence of *Sema-1a*. Confocal images of DC2-, DP1m- and VL2p-adPNs and VA5- and VA7m-lPNs (green; labeled by GAL4-GH146) were used to reveal their dendritic morphologies in the AL. (A, C, E, G and I) No obvious dendritic phenotype was observed in the wild-type DC2-, DP1m- and VL2p-adPNs and VA5- and VA7m-lPNs. We noted that the DC2 adPNs occasionally projected a few dendritic arbors around the VA6 glomerulus (asterisk in panel A and its inset). (B, D, H and J) Dendrites of DC2- and DP1m-adPNs and VA5- and VA7m-lPNs often mis-targeted to the region dorsal to the VC1 glomerulus in the *Sema-1a*^*P1*^ mutant (arrowheads). (F) *Sema-1a*^*P1*^ VL2p adPNs mis-projected their dendrites into the SEZ (arrows) and medially to the region posterior to the VC1 glomerulus (arrowheads in panel F and its inset). (K and L) Two embryonic-born *Sema-1a*^*P1*^ adPNs with the unknown identity (Emb-born (DA3/VC1) adPNs) mis-targeted their dendrites to the DA3 glomerulus and the central portion of the AL in regions close to the VC1 glomerulus. Insets within all panels were derived from single sections of the corresponding confocal images to highlight points of interest. Brain neuropiles (shown in blue) were stained with antibody against Brp. Scale bar: 10 μm.

When we analyzed the phenotypes of those PNs that mis-targeted their dendrites into the DA3 glomerulus, we observed that *Sema-1a*^*P1*^ DC1 adPN dendrites were also mis-projected to a region close to the VC1 glomerulus (100%, n = 3; Fig 2P and its inset; Table 2). This observation led us to look for additional select AL regions (besides the DA3 glomerulus) that could attract dendrites from different sets of PNs when Sema-1a is absent. In our MARCM experiments additional seven types of lPNs and nine types of adPNs were also labeled using GAL4-GH146, allowing us to examine their dendritic patterns (Fig 4). Similar to the DC1 adPN, DC2-, DP1m- and VL2p-adPNs and VA5- and VA7m-lPNs also displayed the phenotype of dendritic mis-targeting to the region around the VC1 glomerulus, with variable mis-projection positions, penetrance and expressivity (31%~87%; Fig 4A–4J; Table 2). Again, over-expressing wild type *Sema-1a* in *Sema-1a*^*P1*^ DC2 adPNs and VA5- and VA7m-lPNs rescued this mis-targeting phenotype: dendrites remained in relatively wild type locations, with dendritic occupancy at the edge and outside of the DC2, VA5 and VA7m glomeruli (S7 Fig; Table 2; similar findings were seen in the DL1 adPNs and DA1- and DL3-lPNs, see S6F, S6I and S8C Figs). The possibility of there being multiple select AL regions that attract dendrites in the absence of *Sema-1a* was further strengthened by our observation of another striking dendritic mis-targeting phenotype, in which dendrites mis-projected into the DA3 glomerulus and into regions in close proximity to the VC1 glomerulus when *Sema-1a* was mutated in two embryonic-born adPNs (Fig 4K and 4L; Tables 1 and 2; the only wild-type DA3 adPNs labeled by GAL4-GH146 are larval-born [6]). Moreover, the VM7v and VA4 glomeruli and the region ventral to the DP1m glomerulus were also prone to aberrant dendritic invasion by DM1-, DM2- and VA7m-lPNs and DL1-, DL5- and DM3-adPNs (S8B and S9 Figs; S4 Table; VM7v adPNs are the only PNs labeled by GAL4-GH146 that innervate the VM7v glomerulus [6]). However, whether the VM7v and VA4 glomeruli and the region ventral to the DP1m glomerulus behave as the select AL regions to attract other PN dendrites remains unclear and awaits the characterization of dendritic patterns for the rest of the PNs that we did not examine here. In contrast, VA2-, VA3- and VM3-adPNs and VA4-, VC1- and VC2-lPNs did not exhibit specific dendritic mis-targeting phenotypes when *Sema-1a* was absent (S1 Table). We also noted that many embryonic-born, and a few larval-born, *Sema-1a*^*P1*^ adPNs/lPNs mis-projected their dendrites into the SEZ without AL innervation, which we never observed in the wild-type samples (S10 Fig; S5 Table). Taken together, these results suggest that one additional select AL region (i.e., the region close to the VC1 glomerulus) may co-exist along with the DA3 glomerulus and that they serve to attract PN dendrites when Sema-1a is absent.

**Table 2:** Phenotypic description of PNs in *wild-type*, *Sema-1a LOF* and rescue experiments in Figs 2 and 4 and S7 Fig.

*Sema-1a*-deficient PNs with a phenotype of dendritic mis-targeting to the region close to the VC1 glomerulus	type of PN (emb/larval)^a^	conditions of Sema-1a expression	total number (n)	% of dendritic mis-targeting in close proximity of the VC1 glomerulus	% of dendritic occupancy in Brp negative regions and/or partial glomerulus	% of other dendritic mis-targeting	notes
	DC1 adPN^e^ (emb; Fig 2)	*wt*	5	0%	0%	20%^d^	^d^ some dendritic arbors around the D glomerulus
		*Sema-1a*^*P1*^	3	100%^b^	0%	67%^d^	^b^ dendritic innervation to the region dorsal to the VC1 glomerulus ^d^ dendritic innervation in the ventrolateral part of the DA3 glomerulus
	DP1m adPN (emb; Fig 4)	*wt*	24	0%	0%	17%^d^	^d^ a patch of dendritic arbors in the DM3 (9%) and DM1 (4%) glomeruli; heavy dendritic innervation in both DP1m and VL2p glomerulus
		*Sema-1a*^*P1*^	34	71%^b^	0%	62%^d^	^b^ dendritic innervation to the region around the VC1 glomerulus ^d^ dendritic innervation in the DM3 glomerulus (56%) and the region lateral to the VM7 glomerulus (26%)
	VL2p adPN (emb; Fig 4)	*wt*	43	5%^b^	0%	9%^d^	^b^ a patch of dendritic arbors ventrally around the VC1 glomerulus ^d^ some dendritic arbors posterior to DP1m, V or VL1 glomerulus or posterior to the AL
		*Sema-1a*^*P1*^	15	87%^b^	0%	87%^d^	^b^ dendritic innervation mostly to the region posterior to the VC1 glomerulus (13% out of 87% without the SEZ innervation) ^d^ dendritic innervation to the SEZ (13% out of 87% without the innervation around the VC1 glomerulus)
	DC2 adPN (larval; Fig 4 & S7)	*wt*	5	0%	0%	40%^d^	^d^ a patch of dendritic arbors around the VA6 glomerulus
		*Sema-1a*^*P1*^	21	45%^b^	0%	32%^d^	^b^ dendritic innervation mostly to the region dorsal to the VC1 glomerulus ^d^ dendritic arbors around and/or within the VA6 glomerulus
		*Sema-1a*^*P1*^+Sema-1a	5	0%	100%^c^	0%	^c^ dendritic innervation largely outside of the DC2 glomerulus (Brp negative region)
	VA5 lPN (larval; Fig 4 & S7)	*wt*	7	0%	0%	0%	
		*Sema-1a*^*P1*^	13	31%^b^	0%	23%^d^	^b^ dendritic innervation to the region ventral to the VC1 glomerulus ^d^ dendritic innervation in part of the VA6 and/or DC3 glomeruli
		*Sema-1a*^*P1*^+Sema-1a	8	0%	100%^c^	0%	^c^ dendritic innervation largely outside of the VA5 glomerulus (Brp negative region)
	VA7m lPN^e^ (larval; Fig 4 & S7)	*wt*	4	0%	0%	25%^d^	^d^ a small patch of dendritic innervation to the VA4 glomerulus
		*Sema-1a*^*P1*^	9	44%^b^	0%	67%^d^	^b^ dendritic innervation to the region close to the VC1 glomerulus ^d^ dendritic innervation in part of the VA4 and/or VC2 glomeruli
		*Sema-1a*^*P1*^+Sema-1a	15	0%	100%^c^	0%	^c^ dendritic innervation largely outside of the VA7m glomerulus (Brp negative region)
	DA3/VC1 adPN^e^ (emb; Fig 4)	*Sema-1a*^*P1*^	2	100%^b^	0%	100%^d^	^b^ dendritic innervation to the region around the VC1 glomerulus ^d^ heavy dendritic accumulation in the DA3 glomerulus

^**a**^ emb:embryonic-born; larval: larval-born.

^**b, c, d**^ see the description in the column of notes.

^**e**^ same types of PNs can be also seen in different classes of dendritic mis-targeting phenotypes in Table 1 and S4 and S5 Tables.

## Discussion

Secreted ligands and cell surface molecules act in concert to regulate the cell-morphogenetic and neurite-sorting processes that generate appropriate patterns of axonal branches and dendritic arbors in neurons of the olfactory system, resulting in constructing the complex, functional olfactory circuitry [18, 19]. In the present study, we discover a new role of Sema-1a to prevent dendrites of adPNs, lPNs and vPNs from aberrantly invading into the select AL regions, which is crucial for generating appropriate discrete PN dendritic patterns to construct the olfactory map within the AL.

**Fig 5:**
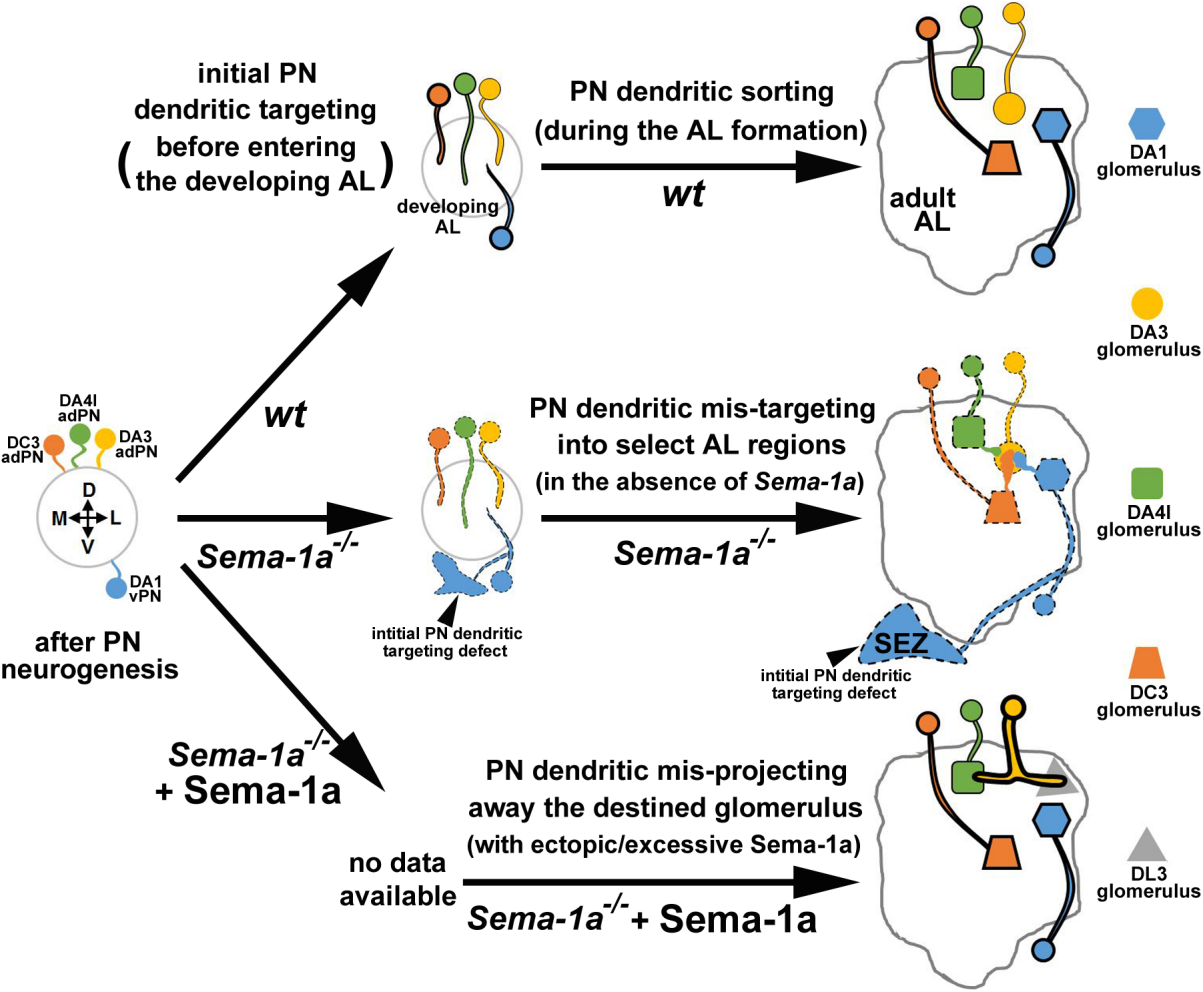
Schematic depiction of how Sema-1a regulates appropriate PN dendritic patterns in the developing AL. After the birth of PNs (PN neurogenesis), the repulsive Sema-1a signal plays an essential role in PNs to determine whether PN dendrites reside within the developing AL in the initial PN dendritic targeting step and PN dendrites often mistakenly invade the region ventromedial to the developing AL (and SEZ; arrowheads) in the absence of *Sema-1a* (from solid-outline wild-type PNs to dash-outline *Sema-1a-*deficient PNs). Once entering the developing AL, dendrites of different types of PNs (e.g., DA3-, DA4l and DC3-adPNs and DA1 vPN) in wild-type animals normally stay away from each other in order to sort into the destined glomeruli (the round (e.g., DA3), square (e.g., DA4l), trapezoid (e.g., DC3) and hexagonal (e.g., DA1) glomeruli, respectively). The repulsive Sema-1a signal may be differentially transmitted in various types of PNs: high (e.g., DC3 adPN and DA1 vPN; thick solid-outline), moderate (e.g., DA4l adPN; less thick solid-outline) and low (e.g., DA3 adPN; thin solid-outline). In the absence of the Sema-1a repulsion, dendrites of *Sema-1a*^*P1*^ PNs (e.g., DA4l- and DC3-adPNs and DA1 vPN) invade select AL regions (e.g., the DA3 glomerulus), resulting in the dendritic mis-targeting phenotypes. In contrast, ectopic and excessive expression of the repulsive Sema-1a signal in the *Sema-1a*^*P1*^ DA3 adPN (very thick solid-outline) drives their dendrites into adjacent glomeruli (e.g., mis-projection of the dendrites of the *Sema-1a*^*P1*^ DA3 adPN into the square (e.g., DA4l) glomerulus and the triangular (e.g., DL3) glomerulus).

Construction of the *Drosophila* adult AL is a complex process to integrate neurites of multiple populations of PNs, LNs and OSNs during the pupal stage [8, 20]. What are the roles of specific molecules in this complex process of neurite sorting and integration among PNs, LNs and OSNs? Sema-1a was proposed to control initial dendritic targeting of PNs along the DL-to-VM axis of the AL based on observations of dorsolateral-enriched expression of Sema-1a in the developing AL and mis-targeting of *Sema-1a*^*P1*^ DL1 adPN dendrites into the region ventromedial to the developing AL (Fig 4C of the Komiyama study) [12]. Interestingly, we observed similar phenotypes in *Sema-1a*^*P1*^ adPNs and lPNs, in which their dendrites mis-projected into the SEZ, a neuropile ventromedial to the adult AL, with or without entering the AL (Figs 1E and 4F; S5B–S5D Fig, S6E and S10 Figs). Therefore, the repulsive Sema-1a signal does play an essential role in the step of initial dendritic targeting by preventing PN dendrites from mistakenly invading the region ventromedial to the AL (e.g., the SEZ; also see the schematic drawing in Fig 5).

The above described step of initial dendritic targeting directed by Sema-1a was further proposed to link with the later refinement and sharpening of boundaries among glomeruli through intercellular interactions, for example dendrite–dendrite interactions among PNs mediated by N-cadherin [12, 21]. A correlated range of severity of the DL-to-VM dendritic shift phenotypes found in *Sema-1a*^*P1*^ PNs—in which DL1 adPNs (having the farthest dorsolateral dendrites) displayed the most severe DL-to-VM dendritic shift compared to the moderate and mild phenotypes of the dendrites of DA1 lPNs and DC3 adPNs—supported the idea that the distribution of PN dendrites in the AL is determined by the Sema-1a expression gradient [12]. Although we observed similar DL-to-VM dendritic shift defects in *Sema-1a*^*P1*^ DL1 adPNs and DA1 lPNs (S6D and S8B Figs), these phenotypes may be also interpreted as mis-targeting of dendrites of DL1 adPNs and DA1 lPNs into unidentified select AL regions (e.g., the region ventral to the DP1m glomerulus for DL1 adPNs shown in the inset of S8B Fig) in the absence of *Sema-1a*. In contrast, *Sema-1a*^*P1*^ DC3 adPNs exhibited the DA3-glomerular dendritic mis-targeting defect and did not show the DL-to-VM dendritic shift phenotype in our study (Fig 2G). Upon close examination of two *Sema-1a*^*P1*^ DC3 adPN images from Komiyama et al. (their Fig 3D) [12], we noted that the bottom right image displays a mild dendritic mis-targeting defect, occupying the ventral tip of the DA3 glomerulus, and the bottom left image was horizontally flipped, so it is not likely to be a DC3 adPN. Both DC3 adPN examples shown in the Komiyama study [12], taken together with the DA3-glomerular dendritic mis-targeting defect we observed in DC3 adPNs (Fig 2G) and also in other PN samples (D-, DA4l-, DA4m-, DC1-, DC3-, DL4- and VA1d-adPNs and DA1- and diffuse-vPNs in Figs 1 and 2), complicates the straightforward DL-to-VM dendritic shift model that was proposed in order to account for the function of Sema-1a in the establishment of appropriate dendritic patterns once PN dendrites have projected into the developing AL.

How then does Sema-1a regulate the formation of dendritic patterns once multiple populations of PNs (e.g., adPNs, lPNs and vPNs) have sent their dendrites into the developing AL? We found here that different sets of uni-glomerular- and poly-glomerular PNs among adPNs, lPNs and vPNs mis-targeted their dendrites into select AL regions, including the DA3 glomerulus and a region close around the VC1 glomerulus, when *Sema-1a* was mutated (Figs 1, 2 and 4). Intriguingly, the PNs that displayed the *Sema-1a*-deficient DA3-glomerular dendritic mis-targeting defect seem to associate with their deriving origins, i.e., the occurrence of the dendritic mis-targeting phenotype only in adPNs and vPNs but not lPNs (Figs 1 and 2 and S6 Fig). In the light of these results, it may be reasonable to speculate the presence of sorting centers in the developing AL for controlling dendrites of uni-glomerular- and poly-glomerular-PNs among adPNs, lPNs and vPNs toward their destined glomeruli. Interestingly, dendrites of adPNs and vPNs seem to accumulate in the anterior dorsolateral portion of the developing AL at 24h APF (double-arrowhead in S2C Fig), which maybe correlate with PN dendritic innervation in the DA3 glomerulus and anterodorsal glomeruli of the AL. When the Sema-1a repulsion (as a driving force) is gone, PN dendrites will be trapped in the sorting centers, which results in to dendritic mis-targeting into select AL regions. However, without figuring out the identity of those PN dendrites in the anterior dorsolateral portion of the developing AL (e.g., whether the green adPN dendrites are destined toward the DA3 glomerulus and its surrounding glomeruli, and whether the magenta PN dendrites are derived from vPNs but not from the DL1 adPN; double-arrowhead in S2C Fig), the presence of dendritic sorting centers in the developing AL remains elusive.

No matter whether the dendritic sorting centers exist in the developing AL or not, how does Sema-1a transmit the repulsion signal in PN dendrites to prevent inappropriately mixing together? Previously, the expression of Sema-2a/-2b in the ventromedial corner of the developing AL has been reported as ligands of Sema-1a to regulate the PN dendritic targeting [13]. However, it may take a slightly complicate mechanism to only involve the graded expression of Sema-2a/-2b to elicit the repulsive Sema-1a signal in PNs to avoid dendritic accumulation in select AL regions. Therefore, it is reasonable to speculate the presence of not-yet identified factors which may work in concert with the repulsive Sema-2a/-2b-Sema-1a signal in PNs. Dependent on the distribution of those not-yet identified factors in the developing AL, various types of uni-glomerular- and poly-glomerular-PNs among adPNs, lPNs and vPNs may interpret the repulsive Sema-1a signal instructively or permissively to generate distinct but overlapping dendritic patterns in discrete glomeruli of the AL. Based on the differing degrees of penetrance and expressivity of the *Sema-1a*-deficient DA3-glomerular dendritic mis-targeting phenotype, we speculate that the repulsive Sema-1a signal may be differentially transmitted in various types of PNs with high (e.g., DC3 adPNs and DA1- and diffuse-vPNs), moderate (e.g., D-, DA4l-, DA4m-, DC1-, DL4- and VA1d-adPNs) to low (e.g., DA3 adPNs) degrees (see Fig 5 for the schematic drawing). Removing Sema-1a from PNs (e.g., DC3- and DA4l-adPNs and DA1 vPNs) that are normally affected by the repulsive Sema-1a signal may turn these *Sema-1a*-deficient PN dendrites to behave more like dendrites of PNs that normally do not respond to the repulsive Sema-1a signal (e.g., DA3 adPNs), which results in dendritic mis-targeting in a wrong AL region (e.g., DC3 and DA4l *Sema-1a*^*P1*^ adPNs and DA1 *Sema-1a*^*P1*^ vPNs mis-target their dendrites into the DA3 glomerulus). On the other hand, excessive and ectopic expression of Sema-1a in the PNs (e.g., DA3 adPNs) that presumably expresses low or no endogenous level of Sema-1a may convert these *Sema-1a*-manipulated PNs into sensitive to the repulsive Sema-1a signal, which results in steering their dendrites away from their destined region (e.g., the DA3 glomerulus) into adjacent glomeruli (e.g., DA4l and DL3 glomeruli; see Fig 5 for the schematic drawing). While our proposed model is able to account for the dendritic mis-targeting phenotypes observed among *Sema-1a*-deficient adPNs, lPNs and vPNs, questions remain concerning how and what not-yet identified factors work together with Sema-1a in various types of PNs to set up correct dendritic patterns. Future identification and investigation of these factors may reveal how extracellular signals that elicit the Sema-1a repulsion in PNs and how Sema-1a works in concert with ensembles of molecules among various types of PNs to generate a precise and functional olfactory map that decodes external olfactory inputs into essential internal information for the survival of animals.

## Materials and methods

### Transgenic construct of UAS-*Sema-1a*^*sy*^

Standard molecular biological techniques were used to generate *UAS-Sema-1a*^*sy*^, in which the *Sema-1a* open reading frame from a *UAS-Sema-1a* cDNA construct [17] was cut with XhoI and XbaI and re-cloned into pJFRC7-20XUAS-IVS-mCD8::GFP [22]. The *UAS-Sema-1a*^*sy*^ construct was injected into a fly stock carrying an attP docking site (VK00033; e.g., Bloomington stock number (BL) 9750) to generate the transgenic fly stock via the service provided by Rainbow Transgenic Flies, Inc.

### Fly strains

The fly strains used in this study were as follows: (1) *hs-FLP*^*122*^ [8]; (2) *FRT40A*,*UAS-rCD2*::*RFP*,*UAS-GFP-miRNA* [15]; (3) *FRT40A*,*UAS-mCD8*::*GFP*,*UAS-rCD2-miRNA*, *GAL4-GH146* [6]; (4) *GAL4-MZ699* [23]; (5) *GH146-FLP* [24]; (6) *UAS-FRT<stop<FRT-myrGFP* [22]; (7) *R95B09-GAL4* (BL47267); (8) *UAS-Sema-1a RNAi*^*TRiP*^ (BL34320); (9) *FRT40A*,*UAS-mCD8*::*GFP*,*GAL4-GH146* [25]; (10) *FRT40A*, *UAS-mCD8*::*GFP*,*Sema-1a*^*k13702*^,*GAL4-GH146* (*Sema-1a*^*k13702*^ is *Sema-1a*^*P1*^ [17] and the original source of *Sema-1a*^*k13702*^ was from Kyoto Stock Center with the stock number 111328); (11) *FRT40A*,*tubP-GAL80* [26]; (12) *UAS-Sema-1a*^*sy*^ (generated in this study); (13) *R38B04-GAL4* (BL49984); (14) *GAL4-MZ19* [27]; (15) *actin-FRT<stop<FRT-GAL4* [2].

### Visualization of wild-type, *Sema-1a* LOF and *Sema-1a* rescued PNs

Flippase-out mediated intersection samples and mosaic clones for the MARCM, flip-out MARCM and twin-spot MARCM studies were generated as previously described [2, 14, 15, 26]. MARCM samples were obtained by collecting embryos in vials and inducing mosaic clones at various developmental periods with heat-shock for 10 to 15 minutes. To pin-point birth-order of PNs in *Sema-1a* LOF and *Sema-1a* rescued MARCM experiments (S2 and S3 Tables), larvae were picked up as NHL to synchronize samples, and mosaic clones were induced at four-hour intervals from 26h ALH to 56h ALH with heat-shock for 10 to 15 minutes. For the flip-out MARCM and twin-spot MARCM experiments, mosaic clones of larval-born adPNs, lPNs, vPNs and lvPNs were generated by heat-shock for 10 to 25 minutes in NHL.

### Fly brain preparation and image processing

Dissection, immunostaining and mounting of more than 5,000 fly brains were performed as described in a standard protocol [26]. Primary antibodies used in this study included rabbit antibody against GFP (1:800, Invitrogen), rabbit antibody against RFP (1:800, Clontech), rat antibody against DN-cadherin (DN-Ex #8, 1:50, DSHB), rat antibody against mCD8 (1:100, Invitrogen), and mouse antibody against Bruchpilot (nc82, 1:50, DSHB). Secondary antibodies conjugated to different fluorophores (Alexa 488, 546, and 647 (Invitrogen)) were used at a 1:800 dilution. Immunofluorescent images of single neurons from single-cell MARCM clones and intersection experiments (around 900 images) and groups of neurons from multi-cellular neuroblast clones of flip-out MARCM and twin-spot MARCM experiments (around 100 images) were collected by Zeiss LSM 700 or 780 confocal microscopy, processed using the Zeiss LSM image browser, and the image intensity adjusted using Photoshop. For the purpose of presentation, wild-type mCD8::GFP- and rCD2::RFP-positive multi-cellular neuroblast clones in the twin-spot MARCM experiments are shown in green for adPNs and lPNs and in magenta for vPNs in S2 Fig. The original LSM files used in the present study are available upon request.

### Quantification of dendritic patterns of DL1 adPNs along the DL-to-VM axis

Scoring of the dendritic patterns of DL1 adPNs was modified from a previous report [12]. Images of interest were projected from confocal stacks containing DL1 adPN dendrites using the Zeiss LSM image browser. Dendritic regions of DL1 adPNs were manually selected and the DL-to-VM axis of the AL was rotated to make it vertical by using Image J. Fluorescent signals were converted into binary numbers using Huang's fuzzy thresholding method provided by Image J [28]. The scoring region was selected and divided into ten bins along the DL-to-VM axis based on Brp-positive staining. The value of the dendritic pattern of DL1 adPNs within the scoring region was summed by Image J. Dendritic intensity and mean position within the scoring bins for dendrites of DL1 adPNs were then calculated. Student’s t-test was used for statistical analysis.

## Supporting information

S1 Fig Dendrites of adPNs+lPNs, vPNs and lvPNs distributed nearly the entire AL. Confocal images of three populations of larval-born PNs (adPNs+lPNs (A), vPNs (B) and lvPNs (C)) were used to display their dendritic occupancy in the adult AL. (A) Dendrites of adPNs (magenta) and lPNs (green) distributed in the adult AL with a non-overlapping fashion. (B and C) Dendrites of vPNs (B) and lvPNs (C) also distributed in the adult AL. We should note that many background neurons (asterisks in the panel C) also existed together with lvPNs due to the utilization of a pan-cell driver in the flip-out MARCM experiment of the panel C. Brain neuropiles (shown in blue) were stained with the antibody against Bruchpilot (Brp). Scale bar: 10 μm.
(TIF)

S2 Fig vPN dendrites migrated anteriorly to mix with dendrites of adPNs and lPNs in the developing AL. Larval-born-adPNs (or lPNs; green arrows) and -vPNs (magenta arrows) were labeled in two distinct colors using GAL4-GH146 and GAL4-MZ699 in the twin-spot MARCM system by simultaneous induction of neuroblast clones at NHL and examining their dendritic patterns at different developmental stages. Three anterior-to-posterior focal sections along the AL axis were shown in twin-spot MARCM clones. (A) Dendrites of adPNs and vPNs occupied dorsal locations in the larval AL (blue arrows) and were segregated at the white pupal stage (WP), in which most of dendrites of adPNs were found anteriorly to those of vPNs. A putative DL1 adPN (light magenta arrowhead) was also found to associate with the green adPNs. (B and C) Segregation of dendrites of adPNs (or lPNs) and vPNs was also observed at 24 hours after puparium formation (24h APF). (D and E) Substantial dendritic mixing between adPNs (or lPNs) and vPNs was observed at 48h APF. (F-H) Dendrites of adPNs (or lPNs) were fully mixed with those of vPNs from 72h APF to the adult AL (blue arrows). Brain neuropiles (shown in blue) were stained with antibodies against Bruchpilot (Brp; A and F-H) or DN-cadherin (DNcad; B-E). Scale bar: 10 μm.
(JPG)

S3 Fig Loss-of-function of *Sema-1a* resulted in dendrites of the DA1 vPN to mis-target to the DA3 glomerulus. (A) A schematic drawing illustrates different dendritic shift defects in DL1 adPNs (brown; dorsolateral-to-ventromedial shift [12]) and DA1 vPNs (green; ventrolateral-to-dorsomedial shift [14]) in *Sema-1a*-loss-of-function (LOF) neurons. D: dorsal, L: lateral, M: medial, V: ventral. (B-G) Individual larval-born DA1 vPNs (green) were labeled using a strategy intersecting R95B09-GAL4 with GH146-FLP (B-E) or the MARCM system with R95B09-GAL4 (F). Confocal images of DA1 vPNs were used to reveal their dendritic patterns in the AL and the subesophageal zone (SEZ). (B) In the wild-type sample, dendrites of the DA1 vPN were predominantly confined within the DA1 glomerulus and not in the DA3 glomerulus. (C) The majority of *Sema-1a* RNAi knock-down samples exhibited the DA3-glomerular dendritic mis-targeting phenotype in DA1 vPNs, in which dendrites significantly invaded into the DA3 glomerulus (85%, n = 49; green signal within the dashed-circle of panel C). (D and E) Two additional dendritic mis-projection phenotypes were also observed in DA1 vPNs within *Sema-1a* RNAi knock-down samples: both phenotypes displayed an aberrant neurite projection to the SEZ (arrows) and no dendritic innervation into the DA1 glomerulus (15%, n = 49; within this class of the phenotype, two additional phenotypes can be further sub-divided into with and without extra dendritic mis-targeting to the DA3-glomerulus in panels D and E (6% and 9%, respectively)). (F) A similar DA3-glomerular dendritic mis-targeting phenotype was also observed when *Sema-1a* RNAi was expressed in the DA1 vPN using the MARCM system (100%, n = 35; green signal within the dashed-circle of panel F). Brain neuropiles (shown) in blue were stained with antibody against Brp and the boundaries of the DA1 and DA3 glomeruli were marked with circles and dashed-circles, respectively, in panels B-F. (G) Percentage of dendritic phenotypes of wild-type and *Sema-1a* RNAi knock-down DA1 vPNs illustrated in panels B-F were shown in the bar graph. The vertical and horizontal axes indicate three types of samples with their examined sample sizes (n) and their phenotypic percentage, respectively. Scale bar: 10 μm.
(TIF)

S4 Fig No DA3-glomerular dendritic mis-targeting phenotype in *Sema-1a^P1^* VA1lm and VL1 vPNs. (A-F) Confocal images of VA1lm and VL1 vPNs (green; labeled by GAL4-GH146) were used to reveal their dendritic morphology in the AL. No DA3-glomerular dendritic mis-targeting phenotype was observed in VA1lm and VL1 vPNs for all three different genotypes: wild-type (A and D), *Sema-1a*^*P1*^ mutant (B and E) and rescued samples of the *Sema-1a*^*P1*^ mutant with *Sema-1a* over-expression (C and F). We noted that a single VA1lm vPN did not occupy the entire VA1lm glomerulus (A-C). Interestingly, dendrites of the wild-type VA1lm vPNs were observed to distribute at the VA1lm glomerulus in different patterns: medially, laterally, in the center and as two splitting aggregates (a wild-type example of two splitting aggregates to occupy the lateromedial and lateral portions of the VA1lm glomerulus was shown in panel A). However, the *Sema-1a*^*P1*^ VA1lm vPNs tended to primarily distribute their dendrites in the medial corner of the VA1lm glomerulus (B). Samples in panels A, B and C were mounted slightly different, which made the dorsal AL glomeruli more prominent and the distance between the DA3 and VA1lm glomeruli longer in panel A. The sexually dimorphic VA1lm glomeruli were also observed in panels A (male), B (male) and C (female) [29]. Brain neuropiles (shown in blue) were stained with antibody against Brp. The boundary of the DA3 and VA1lm glomeruli was marked with dashed-circles and circles in all panels and panels A-C, respectively. Scale bar: 10 μm.
(TIF)

S5 Fig Embryonic-born DA4m adPNs mis-targeted their dendrites into the DA3 glomerulus and SEZ in the *Sema-1a^P1^* mutant. Confocal images of DA4m adPNs (green; labeled by R38B04-GAL4) were used to reveal their dendritic morphology in the AL and the SEZ. (A) In the wild-type sample, dendrites of the DA4m adPNs were largely restricted within the DA4m glomerulus without innervation to the DA3 glomerulus. The green signal within the dashed-circle of panel A was derived from few DA4m adPN dendrites that innervated the posterior AL, which was not observed in confocal sections only covering the depth of the DA3 glomerulus (inset of the panel A). (B-D) Three examples of *Sema-1a*^*P1*^ DA4m adPNs were used to illustrate various dendritic phenotypes: all of them exhibited dendritic innervation in the SEZ (arrows in B-D); for the dendritic phenotypes in the AL, one example had normal DA4m-glomerular dendritic innervation (no green signal within the dashed-circle in B) but showed aberrant dendritic innervation in the ventroposterior AL (arrowheads in B and a single confocal section in inset of the panel B). The other two samples exhibited defects of aberrant dendritic mis-targeting to the DA3 and DA4l glomeruli, which are clearly seen in single confocal sections in insets of the panels C and D. Brain neuropiles (shown in blue) were stained with antibody against Brp, and the boundary of the DA3 glomerulus was marked with dashed-circles in all panels. Scale bar: 12 μm for panel B; 10 μm for panels A, C and D.
(TIF)

S6 Fig DA1 and DL3 lPNs did not display the DA3-glomerular dendritic mis-targeting defect in the *Sema-1a^P1^* mutant. Confocal images of DA1 and DL3 lPNs (green; labeled by GAL4-GH146) were used to reveal their dendritic morphologies in the AL and the SEZ. (A-E, G and H) No DA3-glomerular dendritic mis-targeting defect was observed in wild-type and *Sema-1a*^*P1*^ DA1-and DL3-lPNs. There were three classes of dendritic patterns in the wild-type DA1 lPNs: full (55%; panel A), ventral (29%; panel B) and dorsal (16%; panel C) DA1-glomerular innervation. In contrast, most *Sema-1a*^*P1*^ DA1 lPNs distributed their dendrites to occupy dorsally within the AL (89%; panel D) while the rest of them innervated their dendrites in the whole AL (11%; panel E). Some of *Sema-1a*^*P1*^ DA1 lPNs also sent out neurites to the SEZ (19%; arrows in E). In addition, the DL-to-VM dendritic shifting phenotype was occasionally observed in *Sema-1a*^*P1*^ DA1 lPNs (arrowheads in D), which is consistent with the previous report [12]. (F and I) When Sema-1a was expressed in *Sema-1a*^*P1*^ DA1 and DL3 lPNs, their dendrites occupied the ventral portion of the DA1 glomerulus and were repelled out of the DA1 and DL3 glomeruli to occupy the Brp-negative region around the DA1 and DL3 glomeruli (100%). Two confocal sections were used to represent the dendritic distribution of DL3 lPNs (green signal in the bottom inset of panel I) in the region lacking Brp staining (arrowheads in insets of the panel I). Brain neuropiles (shown in blue) were stained with antibody against Brp, and the boundaries of the DA1 and DA3 glomeruli were marked with circles and dashed-circles, respectively. Scale bar: 10 μm.
(TIF)

S7 Fig Dendrites of DC2 adPNs and VA5- and VA7m-lPNs primarily distributed in the Brp-negative area outside of the glomerulus in the *Sema-1a^P1^* mutant with *Sema-1a* over-expression. Confocal images of DC2 adPNs and VA5- and VA7m-lPNs (green; labeled by GAL4-GH146) were used to reveal their dendritic patterns in the AL. (A-C) Dendrites of DC2 adPNs and VA5- and VA7m-lPNs distributed in areas in proximity to the DC2, VA5 and VA7m glomeruli, respectively, in the *Sema-1a*^*P1*^ mutant with *Sema-1a* over-expression. However, close examination of the dendritic distribution of these PNs revealed their primary occupancy in Brp-negative regions. Two confocal sections were used to represent the dendritic distribution of DC2 adPNs and VA5- and VA7m-lPNs (green signal in bottom insets of the panels A-C) in the regions lacking Brp staining (arrowheads in insets of the panels A-C). Brain neuropiles (shown in blue) were stained with antibody against Brp. Scale bar: 10 μm.
(TIF)

S8 Fig Dendrites of DL1 adPNs underwent a dorsolateral-to-ventromedial shift in the *Sema-1a^P1^* mutant. (A-C) Confocal images of DL1 adPNs (green; labeled by GAL4-GH146) were used to reveal their dendritic patterns in the AL. Compared to dendrites of the wild-type DL1 adPN (A), dendrites of the *Sema-1a*^*P1*^ DL1 adPN tended to undergo a DL-to-VM shift (arrows in B). Single confocal sections of wild-type and *Sema-1a*^*P1*^ animals were used to display the regions of the dendritic mis-targeting defect observed in the *Sema-1a*^*P1*^ DL1 adPN (insets of the panels A and B). *Sema-1a*^*P1*^ DL1 adPNs exhibited a low-penetrant dendritic mis-targeting phenotype (17%; S4 Table) reminiscent of those observed in *Sema-1a*^*P1*^ DL5- and DM3-adPNs, in which dendrites mis-projected into the region ventral to the DP1m glomerulus (arrowheads in S9J and S9L Fig). (C) Manipulation of *Sema-1a* expression in DL1 adPNs by over-expression of *Sema-1a* under the control of GAL4-GH146 in the *Sema-1a*^*P1*^ mutant caused their dendrites to occupy the Brp-negative region outside of the DL1 glomerulus. Two confocal sections were used to show the dendritic distribution of DL1 adPNs (green signal in bottom inset of the panel C) in the region lacking Brp staining (indicated by arrowheads in insets of the panel C). (D and E) Calculation of the DL1 adPN dendritic distribution across the DL-to-VM axis of the AL adapted from the method described previously [12]. The mean position of the *Sema-1a*^*P1*^ DL1 adPN dendrites (2.59±0.45 Bins; n = 35) is significantly different from that of the wild-type DL1 adPN dendrites (1.88±0.26 Bins; n = 12). Our result for the DL1 adPN dendritic distribution within the AL is consistent with previous findings [12]. Brain neuropiles (shown in blue) were stained with antibody against Brp. Scale bar: 10 μm.
(TIF)

S9 Fig Dendrites of DM1-, DM2- and VA7m-lPNs and DM3- and DL5-adPNs tended to mis-project to the VM7v and VA4 glomeruli and the region ventral to the DP1m glomerulus in the *Sema-1a^P1^* mutant. Confocal images of DM1-, DM2- and VA7m-lPNs and DM3- and DL5-adPNs (green; labeled by GAL4-GH146) were used to reveal their dendritic patterns in the AL. (A-D) Dendrites of *Sema-1a*^*P1*^ DM1- and DM2-lPNs were prone to mis-target to the VM7v glomerulus compared to those of wild-type DM1- and DM2-lPNs (arrowheads in B and D). Single confocal sections were used to represent the defect of dendritic mis-targeting to the VM7v glomerulus in wild-type and *Sema-1a*^*P1*^ samples (insets of panels A-D). (E-H) Dendrites of *Sema-1a*^*P1*^ DM1- and VA7m-lPNs also tended to mis-project to the VA4 glomerulus compared to those of wild-type DM1- and VA7m-lPNs (arrowheads in F and H). Single confocal sections were used to represent the defect of dendritic mis-targeting to the VA4 glomerulus in *Sema-1a*^*P1*^ DM1- and VA7m-lPNs (insets of panels F and H). (I-L) *Sema-1a*^*P1*^ DM3- and DL5-adPNs displayed more severe phenotypes of dendritic mis-targeting to the region ventral to the DP1m glomerulus compared to those of wild-type samples (arrowheads in I-L). Single confocal sections were used to represent the defect of dendritic mis-targeting to the region ventral to the DP1m glomerulus in *Sema-1a*^*P1*^ DM3- and DL5-adPNs (insets of panels I-L). Brain neuropiles (shown in blue) were stained with antibody against Brp. Scale bar: 10 μm.
(TIF)

S10 Fig Examples of dendritic mis-targeting to the SEZ in *Sema-1a^P1^* embryonic-born- and larval-born- adPNs and lPNs. Confocal images of embryonic-born (Emb) adPNs and larval-born (Larval) adPNs and lPNs (green; labeled by GAL4-GH146) were used to reveal their dendritic morphologies in the AL and the SEZ. (A-C) Dendritic mis-targeting to the SEZ was found in many embryonic-born *Sema-1a*^*P1*^ adPNs and a few larval-born *Sema-1a*^*P1*^ adPNs and lPNs (arrows). A single *Sema-1a*^*P1*^ DA3 adPN was also observed in panel B (arrowheads). Brain neuropiles (shown in blue) were stained with antibody against Brp. Scale bar: 10 μm.
(TIF)

S1 Table Phenotypic description of PNs in wild-type, Sema-1a LOF and rescue experiments in S4 and S6 Figs. (PDF)

S2 Table Generation of specific types of *Sema-1a^P1^*adPNs in the synchronized MARCM experiment based on their birth-order. (PDF)

S3 Table Generation of specific types of adPNs in the *Sema-1a^P1^* mutant with ectopic *Sema-1a* expression in the synchronized MARCM experiment based on their birth-order. (PDF)

S4 Table Phenotypic description of PNs in wild-type, Sema-1a LOF and rescue experiments in S8 and S9 Figs. (PDF)

S5 Table Phenotypic description of PNs in wild-type, Sema-1a LOF and rescue experiments in Figs 1 and 4 and S5, S6 and S10 Figs. (PDF)

S6 Table Genotypes of the flies in the figure panels. (PDF)
