# Supplementary figures and images for: Semaphorin-1a prevents Drosophila olfactory projection neuron dendrites from mis-targeting into select antennal lobe regions
Source: PLoS Genet. 2017 Apr 27;13(4):e1006751. doi: 10.1371/journal.pgen.1006751 (PMC5426794; doi:10.1371/journal.pgen.1006751)

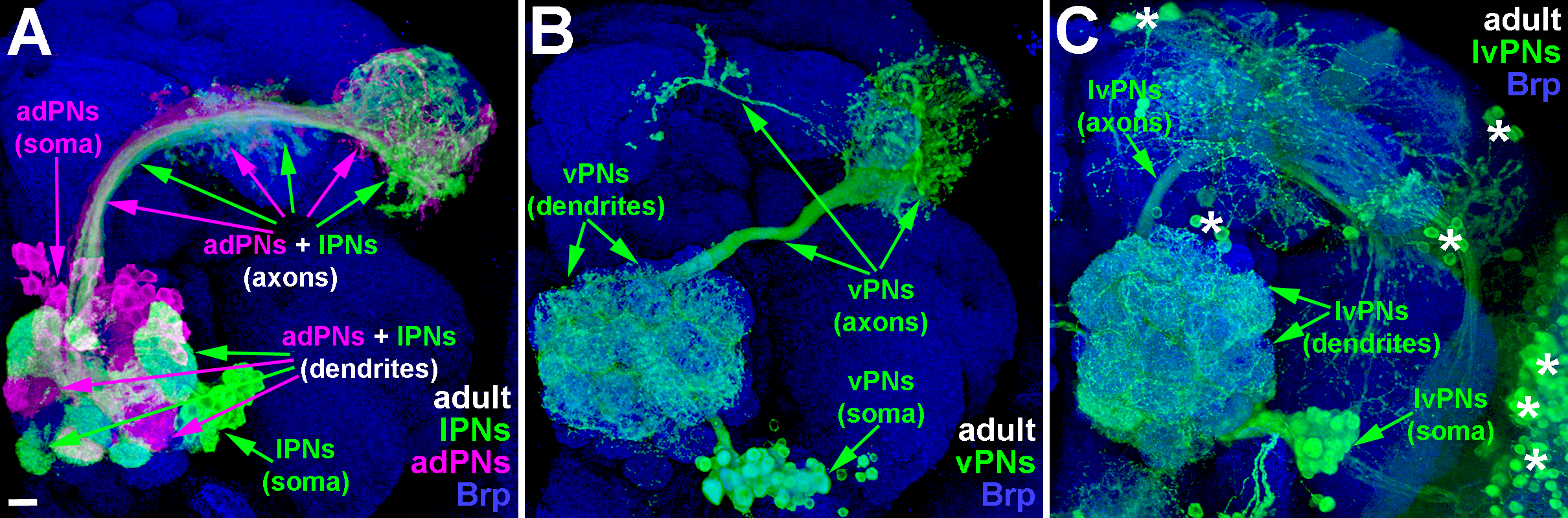

Supplement: S1 Fig — Confocal images of three populations of larval-born PNs (adPNs+lPNs (A), vPNs (B) and lvPNs (C)) were used to display their dendritic occupancy in the adult AL. (A) Dendrites of adPNs (magenta) and lPNs (green) distributed in the adult AL with a non-overlapping fashion. (B and C) Dendrites of vPNs (B) and lvPNs (C) also distributed in the adult AL. We should note that many background neurons (asterisks in the panel C) also existed together with lvPNs due to the utilization of a pan-cell driver in the flip-out MARCM experiment of the panel C. Brain neuropiles (shown in blue) were stained with the antibody against Bruchpilot (Brp). Scale bar: 10 μm. (TIF) [file pgen.1006751.s001.tif]

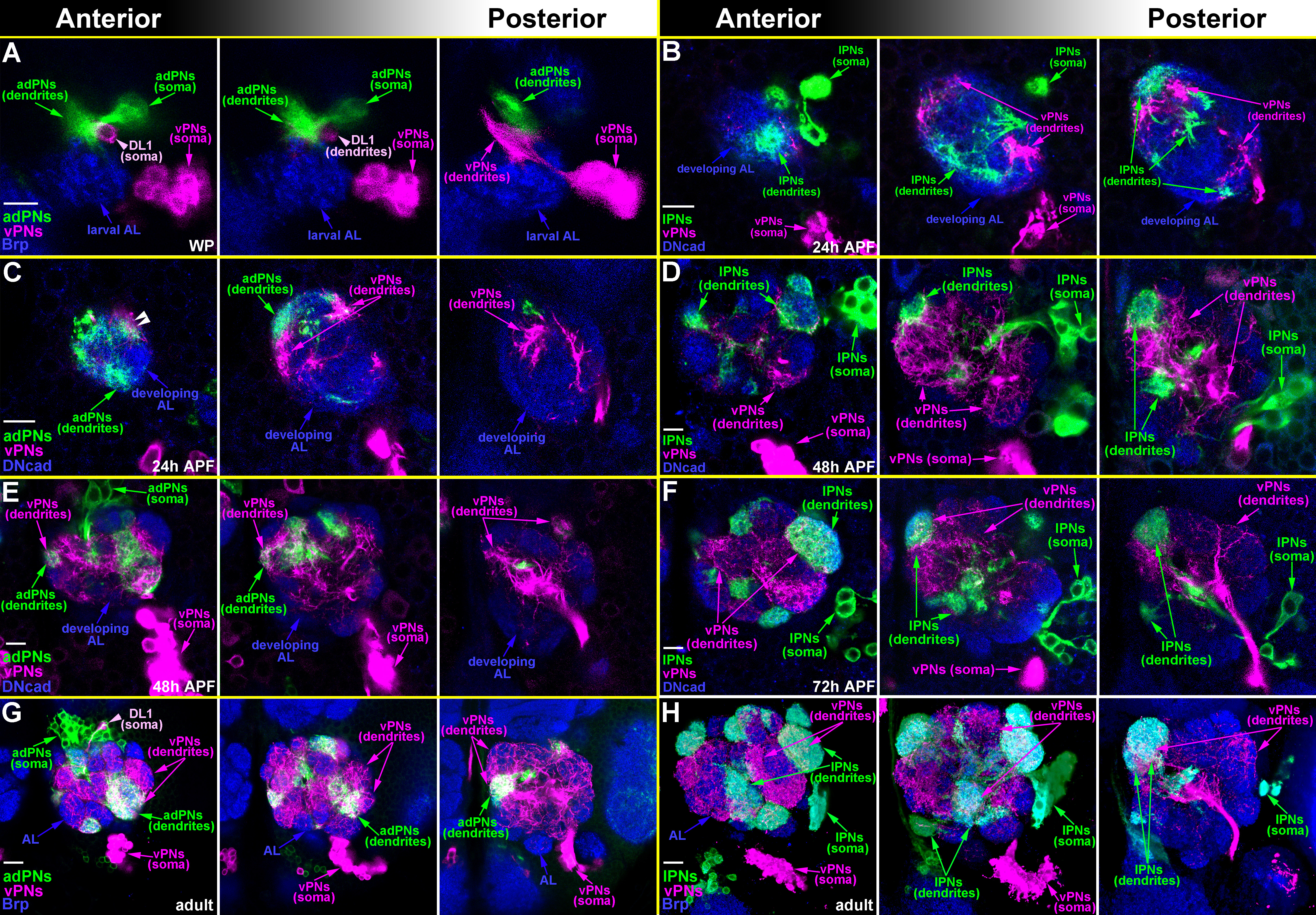

Supplement: S2 Fig — Larval-born-adPNs (or lPNs; green arrows) and -vPNs (magenta arrows) were labeled in two distinct colors using GAL4-GH146 and GAL4-MZ699 in the twin-spot MARCM system by simultaneous induction of neuroblast clones at NHL and examining their dendritic patterns at different developmental stages. Three anterior-to-posterior focal sections along the AL axis were shown in twin-spot MARCM clones. (A) Dendrites of adPNs and vPNs occupied dorsal locations in the larval AL (blue arrows) and were segregated at the white pupal stage (WP), in which most of dendrites of adPNs were found anteriorly to those of vPNs. A putative DL1 adPN (light magenta arrowhead) was also found to associate with the green adPNs. (B and C) Segregation of dendrites of adPNs (or lPNs) and vPNs was also observed at 24 hours after puparium formation (24h APF). (D and E) Substantial dendritic mixing between adPNs (or lPNs) and vPNs was observed at 48h APF. (F-H) Dendrites of adPNs (or lPNs) were fully mixed with those of vPNs from 72h APF to the adult AL (blue arrows). Brain neuropiles (shown in blue) were stained with antibodies against Bruchpilot (Brp; A and F-H) or DN-cadherin (DNcad; B-E). Scale bar: 10 μm. (JPG) [file pgen.1006751.s002.jpg]

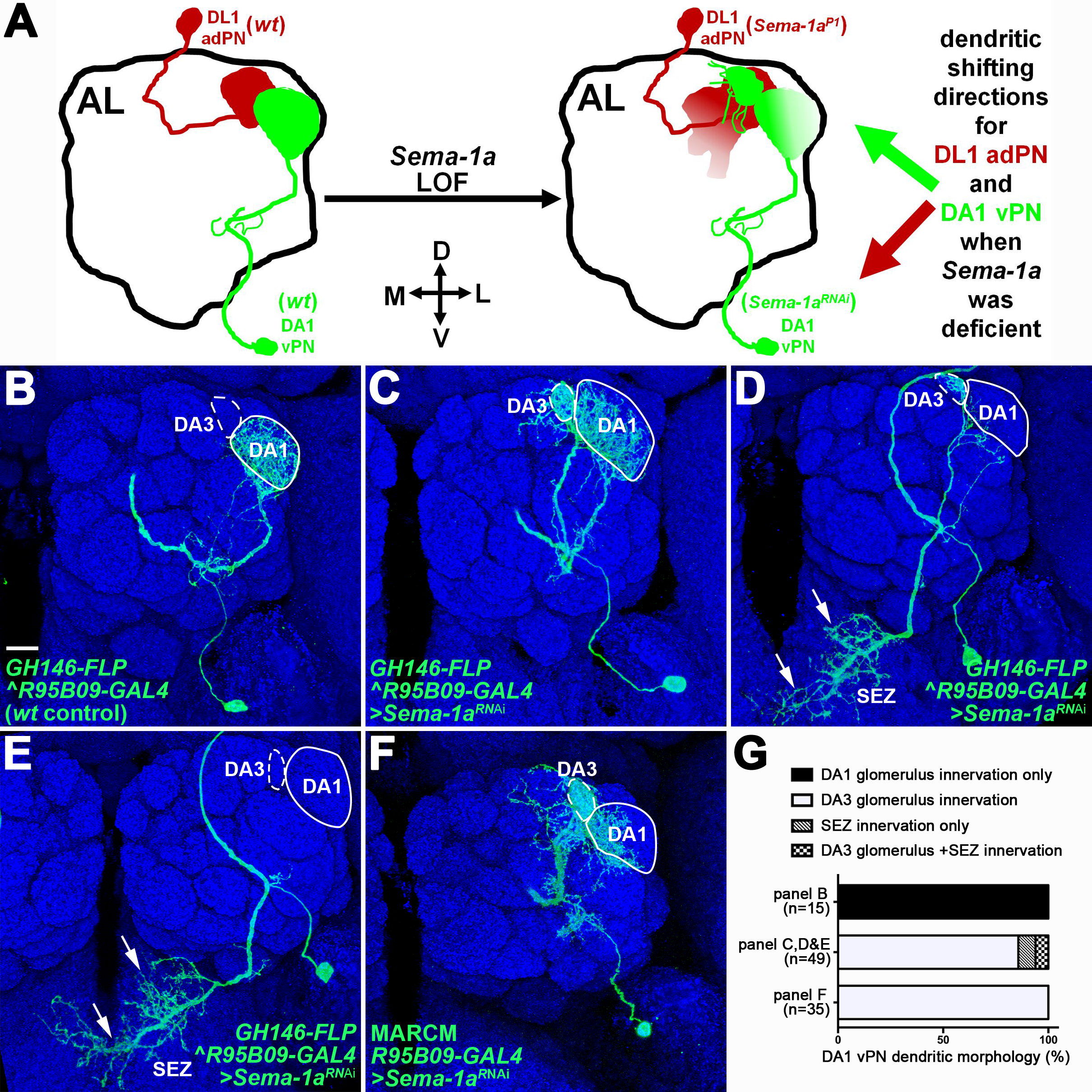

Supplement: S3 Fig — (A) A schematic drawing illustrates different dendritic shift defects in DL1 adPNs (brown; dorsolateral-to-ventromedial shift [12]) and DA1 vPNs (green; ventrolateral-to-dorsomedial shift [14]) in Sema-1a-loss-of-function (LOF) neurons. D: dorsal, L: lateral, M: medial, V: ventral. (B-G) Individual larval-born DA1 vPNs (green) were labeled using a strategy intersecting R95B09-GAL4 with GH146-FLP (B-E) or the MARCM system with R95B09-GAL4 (F). Confocal images of DA1 vPNs were used to reveal their dendritic patterns in the AL and the subesophageal zone (SEZ). (B) In the wild-type sample, dendrites of the DA1 vPN were predominantly confined within the DA1 glomerulus and not in the DA3 glomerulus. (C) The majority of Sema-1a RNAi knock-down samples exhibited the DA3-glomerular dendritic mis-targeting phenotype in DA1 vPNs, in which dendrites significantly invaded into the DA3 glomerulus (85%, n = 49; green signal within the dashed-circle of panel C). (D and E) Two additional dendritic mis-projection phenotypes were also observed in DA1 vPNs within Sema-1a RNAi knock-down samples: both phenotypes displayed an aberrant neurite projection to the SEZ (arrows) and no dendritic innervation into the DA1 glomerulus (15%, n = 49; within this class of the phenotype, two additional phenotypes can be further sub-divided into with and without extra dendritic mis-targeting to the DA3-glomerulus in panels D and E (6% and 9%, respectively)). (F) A similar DA3-glomerular dendritic mis-targeting phenotype was also observed when Sema-1a RNAi was expressed in the DA1 vPN using the MARCM system (100%, n = 35; green signal within the dashed-circle of panel F). Brain neuropiles (shown) in blue were stained with antibody against Brp and the boundaries of the DA1 and DA3 glomeruli were marked with circles and dashed-circles, respectively, in panels B-F. (G) Percentage of dendritic phenotypes of wild-type and Sema-1a RNAi knock-down DA1 vPNs illustrated in panels B-F were shown in the bar graph. [file pgen.1006751.s003.tif]

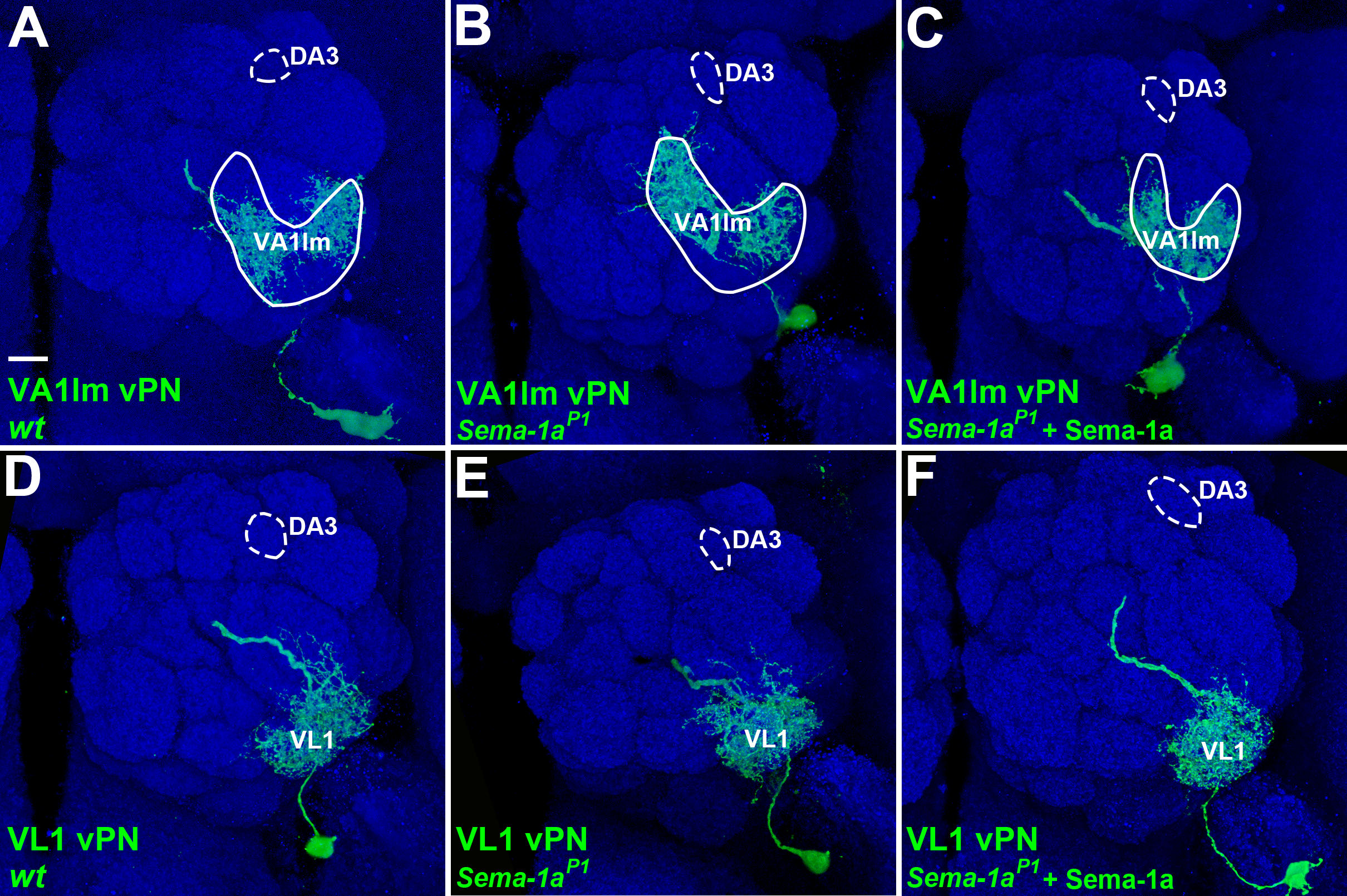

Supplement: S4 Fig — (A-F) Confocal images of VA1lm and VL1 vPNs (green; labeled by GAL4-GH146) were used to reveal their dendritic morphology in the AL. No DA3-glomerular dendritic mis-targeting phenotype was observed in VA1lm and VL1 vPNs for all three different genotypes: wild-type (A and D), Sema-1aP1 mutant (B and E) and rescued samples of the Sema-1aP1 mutant with Sema-1a over-expression (C and F). We noted that a single VA1lm vPN did not occupy the entire VA1lm glomerulus (A-C). Interestingly, dendrites of the wild-type VA1lm vPNs were observed to distribute at the VA1lm glomerulus in different patterns: medially, laterally, in the center and as two splitting aggregates (a wild-type example of two splitting aggregates to occupy the lateromedial and lateral portions of the VA1lm glomerulus was shown in panel A). However, the Sema-1aP1 VA1lm vPNs tended to primarily distribute their dendrites in the medial corner of the VA1lm glomerulus (B). Samples in panels A, B and C were mounted slightly different, which made the dorsal AL glomeruli more prominent and the distance between the DA3 and VA1lm glomeruli longer in panel A. The sexually dimorphic VA1lm glomeruli were also observed in panels A (male), B (male) and C (female) [29]. Brain neuropiles (shown in blue) were stained with antibody against Brp. The boundary of the DA3 and VA1lm glomeruli was marked with dashed-circles and circles in all panels and panels A-C, respectively. Scale bar: 10 μm. (TIF) [file pgen.1006751.s004.tif]

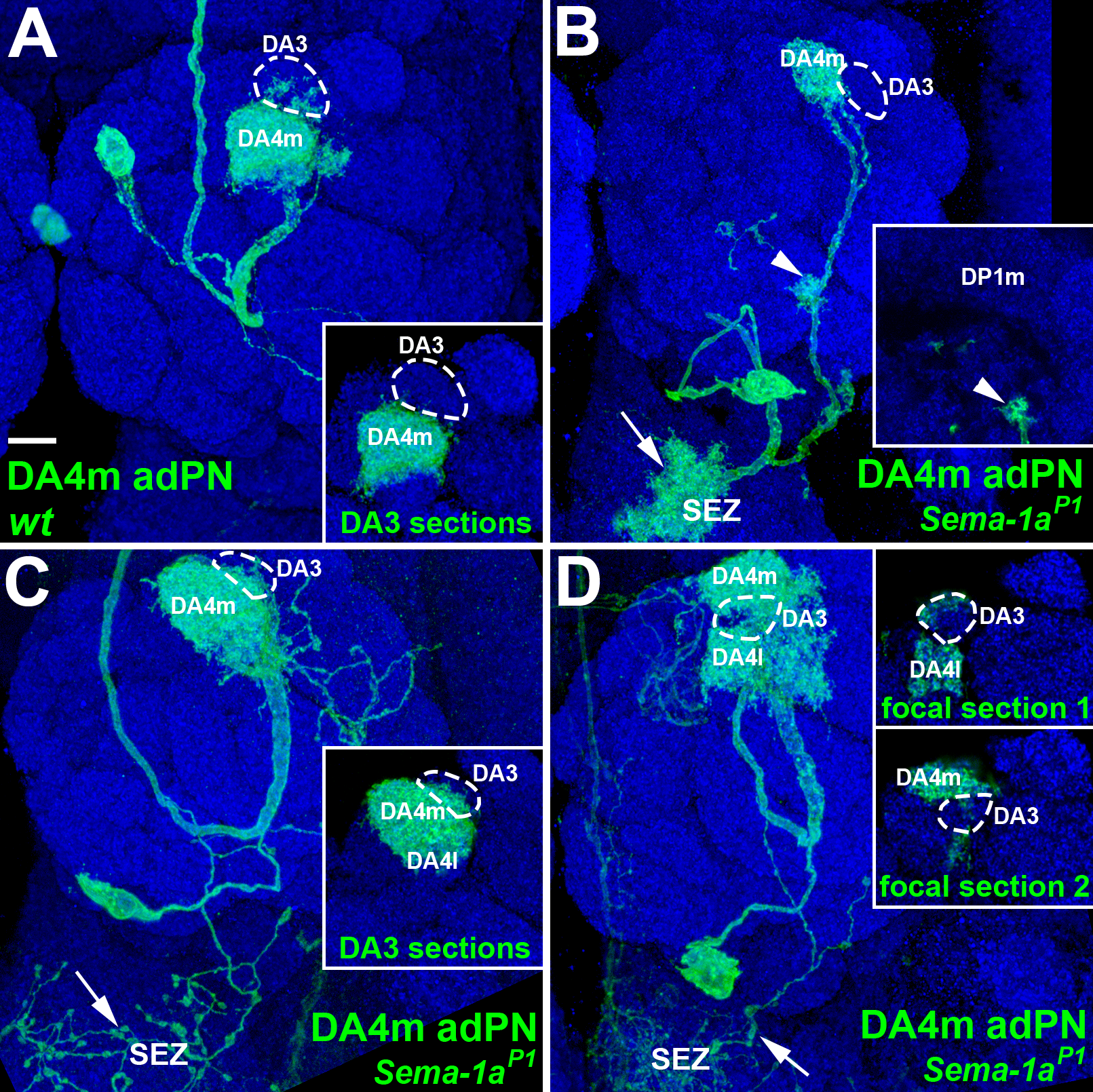

Supplement: S5 Fig — Confocal images of DA4m adPNs (green; labeled by R38B04-GAL4) were used to reveal their dendritic morphology in the AL and the SEZ. (A) In the wild-type sample, dendrites of the DA4m adPNs were largely restricted within the DA4m glomerulus without innervation to the DA3 glomerulus. The green signal within the dashed-circle of panel A was derived from few DA4m adPN dendrites that innervated the posterior AL, which was not observed in confocal sections only covering the depth of the DA3 glomerulus (inset of the panel A). (B-D) Three examples of Sema-1aP1 DA4m adPNs were used to illustrate various dendritic phenotypes: all of them exhibited dendritic innervation in the SEZ (arrows in B-D); for the dendritic phenotypes in the AL, one example had normal DA4m-glomerular dendritic innervation (no green signal within the dashed-circle in B) but showed aberrant dendritic innervation in the ventroposterior AL (arrowheads in B and a single confocal section in inset of the panel B). The other two samples exhibited defects of aberrant dendritic mis-targeting to the DA3 and DA4l glomeruli, which are clearly seen in single confocal sections in insets of the panels C and D. Brain neuropiles (shown in blue) were stained with antibody against Brp, and the boundary of the DA3 glomerulus was marked with dashed-circles in all panels. Scale bar: 12 μm for panel B; 10 μm for panels A, C and D. (TIF) [file pgen.1006751.s005.tif]

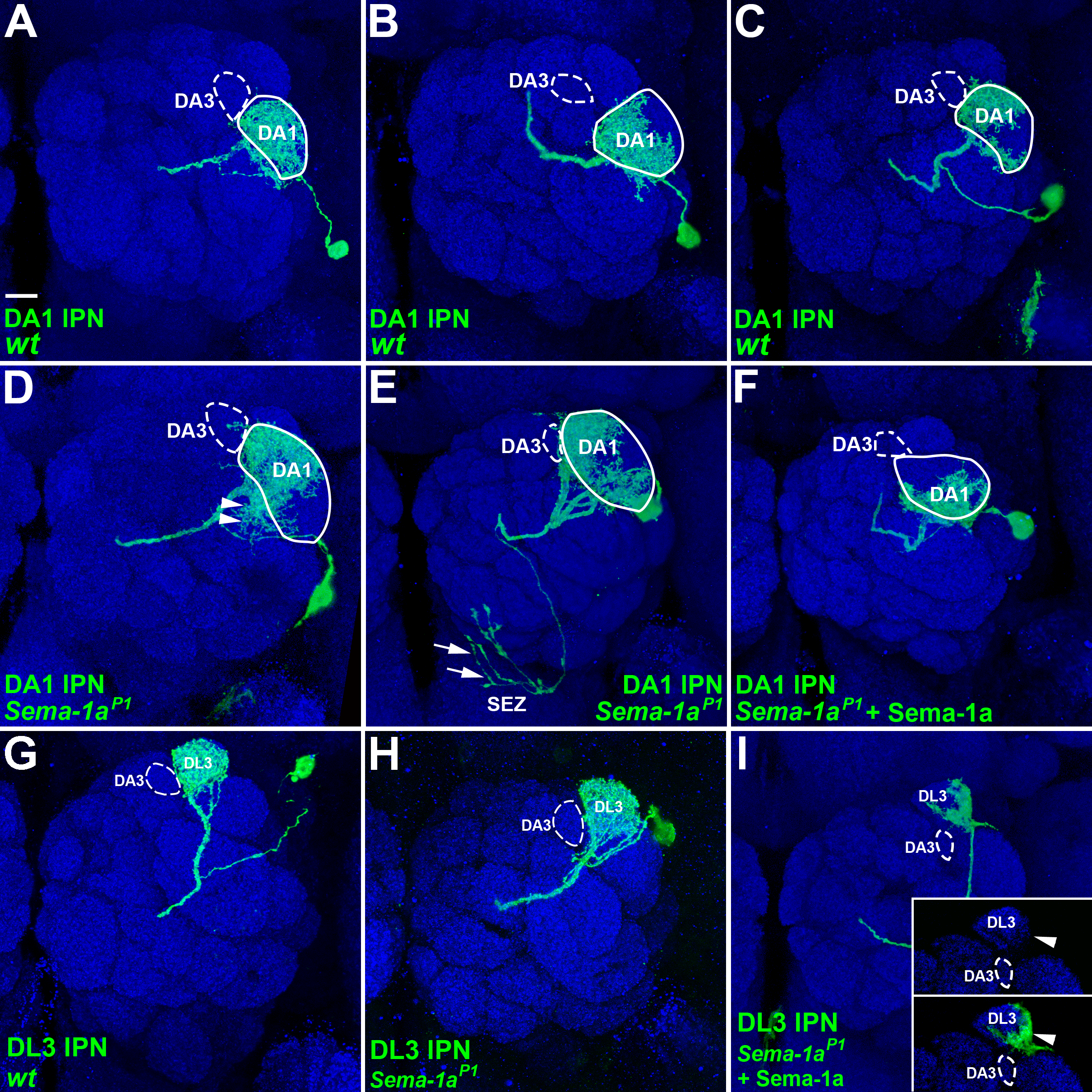

Supplement: S6 Fig — Confocal images of DA1 and DL3 lPNs (green; labeled by GAL4-GH146) were used to reveal their dendritic morphologies in the AL and the SEZ. (A-E, G and H) No DA3-glomerular dendritic mis-targeting defect was observed in wild-type and Sema-1aP1 DA1-and DL3-lPNs. There were three classes of dendritic patterns in the wild-type DA1 lPNs: full (55%; panel A), ventral (29%; panel B) and dorsal (16%; panel C) DA1-glomerular innervation. In contrast, most Sema-1aP1 DA1 lPNs distributed their dendrites to occupy dorsally within the AL (89%; panel D) while the rest of them innervated their dendrites in the whole AL (11%; panel E). Some of Sema-1aP1 DA1 lPNs also sent out neurites to the SEZ (19%; arrows in E). In addition, the DL-to-VM dendritic shifting phenotype was occasionally observed in Sema-1aP1 DA1 lPNs (arrowheads in D), which is consistent with the previous report [12]. (F and I) When Sema-1a was expressed in Sema-1aP1 DA1 and DL3 lPNs, their dendrites occupied the ventral portion of the DA1 glomerulus and were repelled out of the DA1 and DL3 glomeruli to occupy the Brp-negative region around the DA1 and DL3 glomeruli (100%). Two confocal sections were used to represent the dendritic distribution of DL3 lPNs (green signal in the bottom inset of panel I) in the region lacking Brp staining (arrowheads in insets of the panel I). Brain neuropiles (shown in blue) were stained with antibody against Brp, and the boundaries of the DA1 and DA3 glomeruli were marked with circles and dashed-circles, respectively. Scale bar: 10 μm. (TIF) [file pgen.1006751.s006.tif]

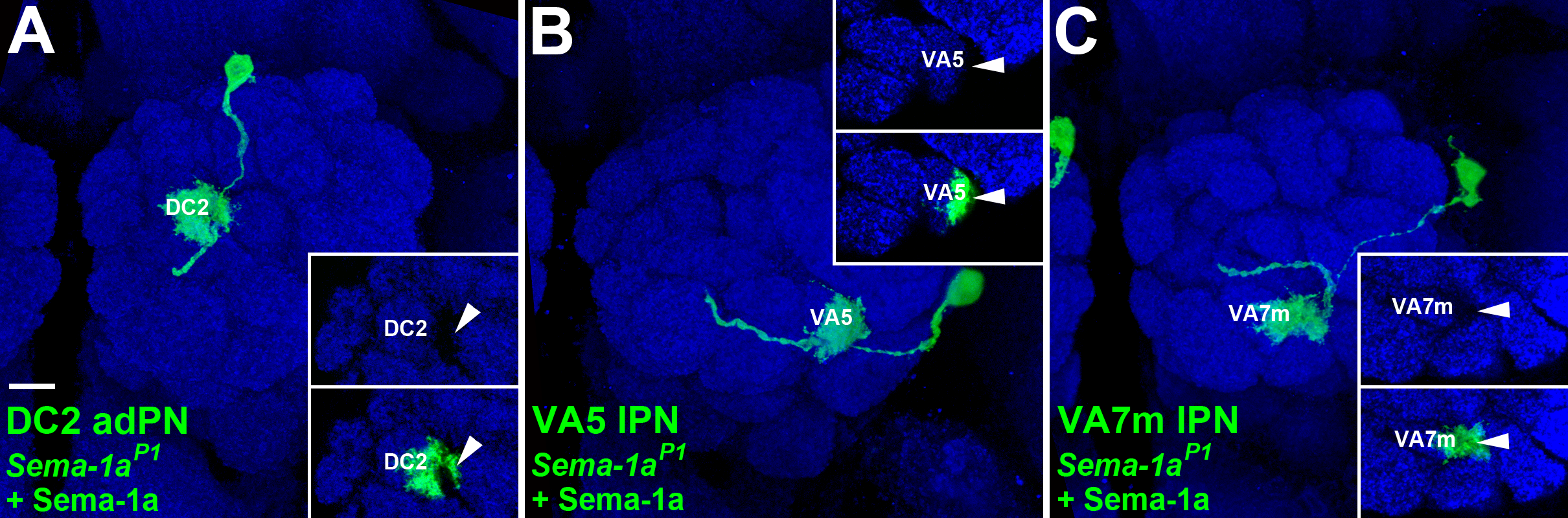

Supplement: S7 Fig — Confocal images of DC2 adPNs and VA5- and VA7m-lPNs (green; labeled by GAL4-GH146) were used to reveal their dendritic patterns in the AL. (A-C) Dendrites of DC2 adPNs and VA5- and VA7m-lPNs distributed in areas in proximity to the DC2, VA5 and VA7m glomeruli, respectively, in the Sema-1aP1 mutant with Sema-1a over-expression. However, close examination of the dendritic distribution of these PNs revealed their primary occupancy in Brp-negative regions. Two confocal sections were used to represent the dendritic distribution of DC2 adPNs and VA5- and VA7m-lPNs (green signal in bottom insets of the panels A-C) in the regions lacking Brp staining (arrowheads in insets of the panels A-C). Brain neuropiles (shown in blue) were stained with antibody against Brp. Scale bar: 10 μm. (TIF) [file pgen.1006751.s007.tif]

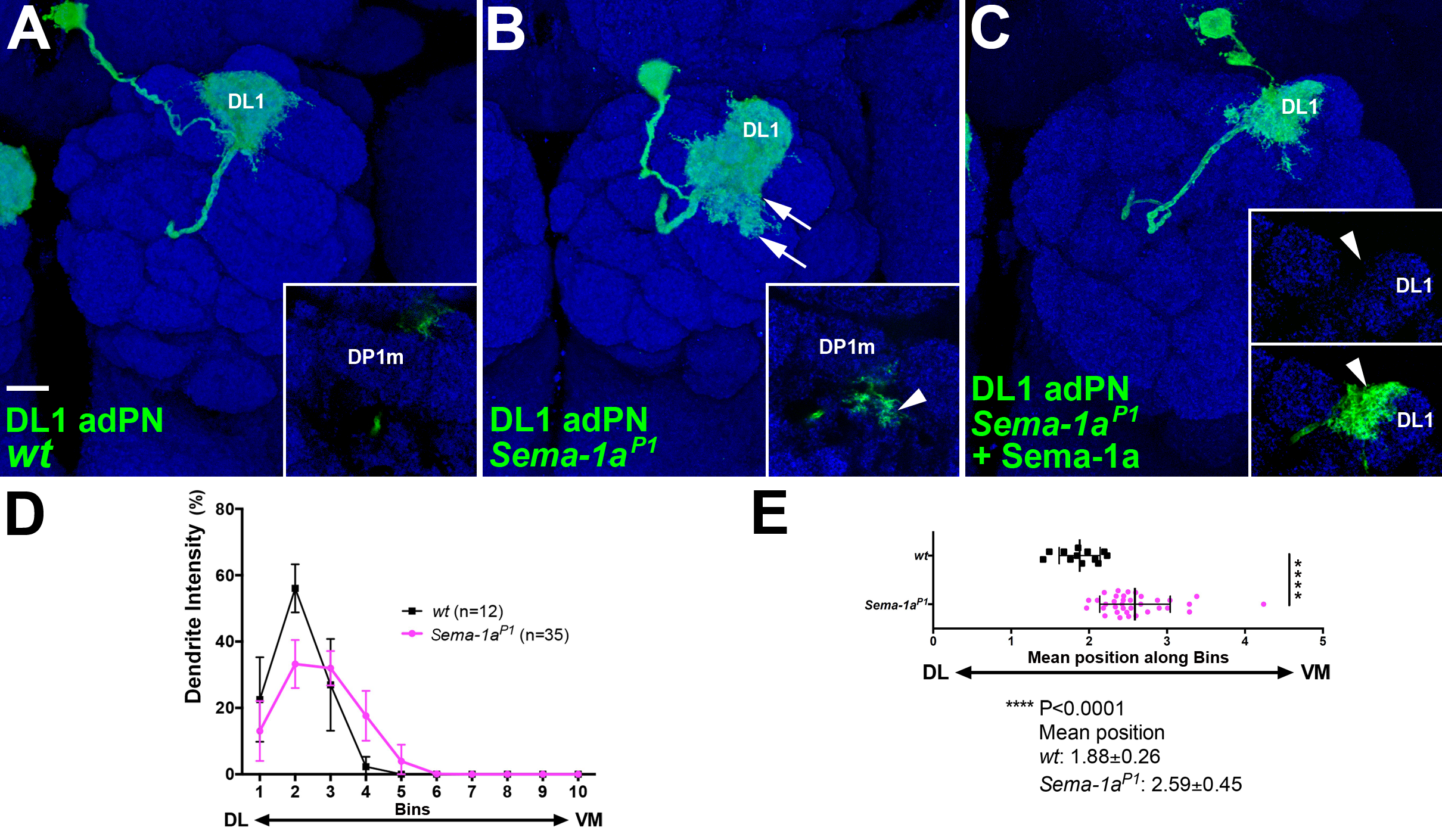

Supplement: S8 Fig — (A-C) Confocal images of DL1 adPNs (green; labeled by GAL4-GH146) were used to reveal their dendritic patterns in the AL. Compared to dendrites of the wild-type DL1 adPN (A), dendrites of the Sema-1aP1 DL1 adPN tended to undergo a DL-to-VM shift (arrows in B). Single confocal sections of wild-type and Sema-1aP1 animals were used to display the regions of the dendritic mis-targeting defect observed in the Sema-1aP1 DL1 adPN (insets of the panels A and B). Sema-1aP1 DL1 adPNs exhibited a low-penetrant dendritic mis-targeting phenotype (17%; S4 Table) reminiscent of those observed in Sema-1aP1 DL5- and DM3-adPNs, in which dendrites mis-projected into the region ventral to the DP1m glomerulus (arrowheads in S9J and S9L Fig). (C) Manipulation of Sema-1a expression in DL1 adPNs by over-expression of Sema-1a under the control of GAL4-GH146 in the Sema-1aP1 mutant caused their dendrites to occupy the Brp-negative region outside of the DL1 glomerulus. Two confocal sections were used to show the dendritic distribution of DL1 adPNs (green signal in bottom inset of the panel C) in the region lacking Brp staining (indicated by arrowheads in insets of the panel C). (D and E) Calculation of the DL1 adPN dendritic distribution across the DL-to-VM axis of the AL adapted from the method described previously [12]. The mean position of the Sema-1aP1 DL1 adPN dendrites (2.59±0.45 Bins; n = 35) is significantly different from that of the wild-type DL1 adPN dendrites (1.88±0.26 Bins; n = 12). Our result for the DL1 adPN dendritic distribution within the AL is consistent with previous findings [12]. Brain neuropiles (shown in blue) were stained with antibody against Brp. Scale bar: 10 μm. (TIF) [file pgen.1006751.s008.tif]

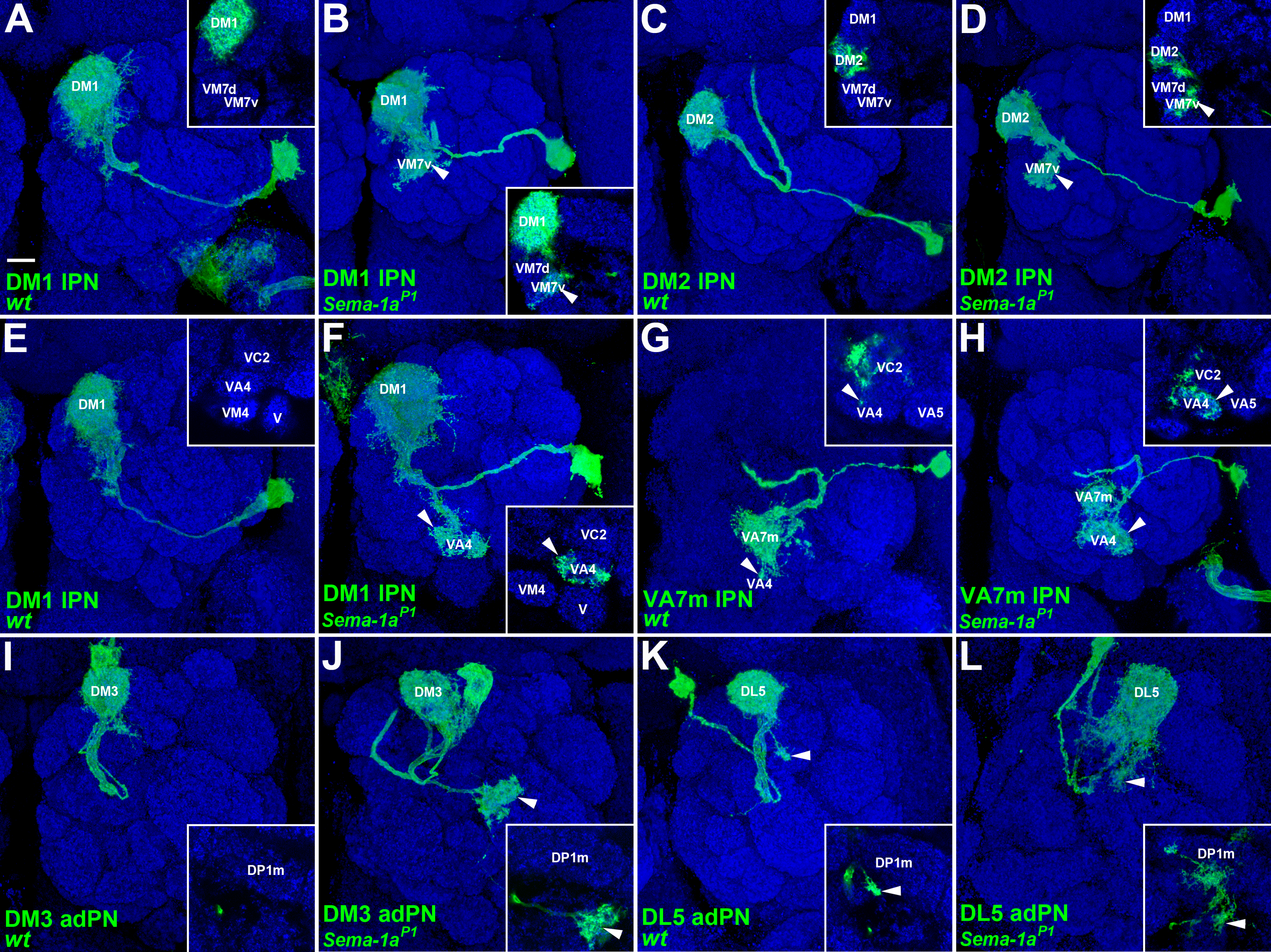

Supplement: S9 Fig — Confocal images of DM1-, DM2- and VA7m-lPNs and DM3- and DL5-adPNs (green; labeled by GAL4-GH146) were used to reveal their dendritic patterns in the AL. (A-D) Dendrites of Sema-1aP1 DM1- and DM2-lPNs were prone to mis-target to the VM7v glomerulus compared to those of wild-type DM1- and DM2-lPNs (arrowheads in B and D). Single confocal sections were used to represent the defect of dendritic mis-targeting to the VM7v glomerulus in wild-type and Sema-1aP1 samples (insets of panels A-D). (E-H) Dendrites of Sema-1aP1 DM1- and VA7m-lPNs also tended to mis-project to the VA4 glomerulus compared to those of wild-type DM1- and VA7m-lPNs (arrowheads in F and H). Single confocal sections were used to represent the defect of dendritic mis-targeting to the VA4 glomerulus in Sema-1aP1 DM1- and VA7m-lPNs (insets of panels F and H). (I-L) Sema-1aP1 DM3- and DL5-adPNs displayed more severe phenotypes of dendritic mis-targeting to the region ventral to the DP1m glomerulus compared to those of wild-type samples (arrowheads in I-L). Single confocal sections were used to represent the defect of dendritic mis-targeting to the region ventral to the DP1m glomerulus in Sema-1aP1 DM3- and DL5-adPNs (insets of panels I-L). Brain neuropiles (shown in blue) were stained with antibody against Brp. Scale bar: 10 μm. (TIF) [file pgen.1006751.s009.tif]

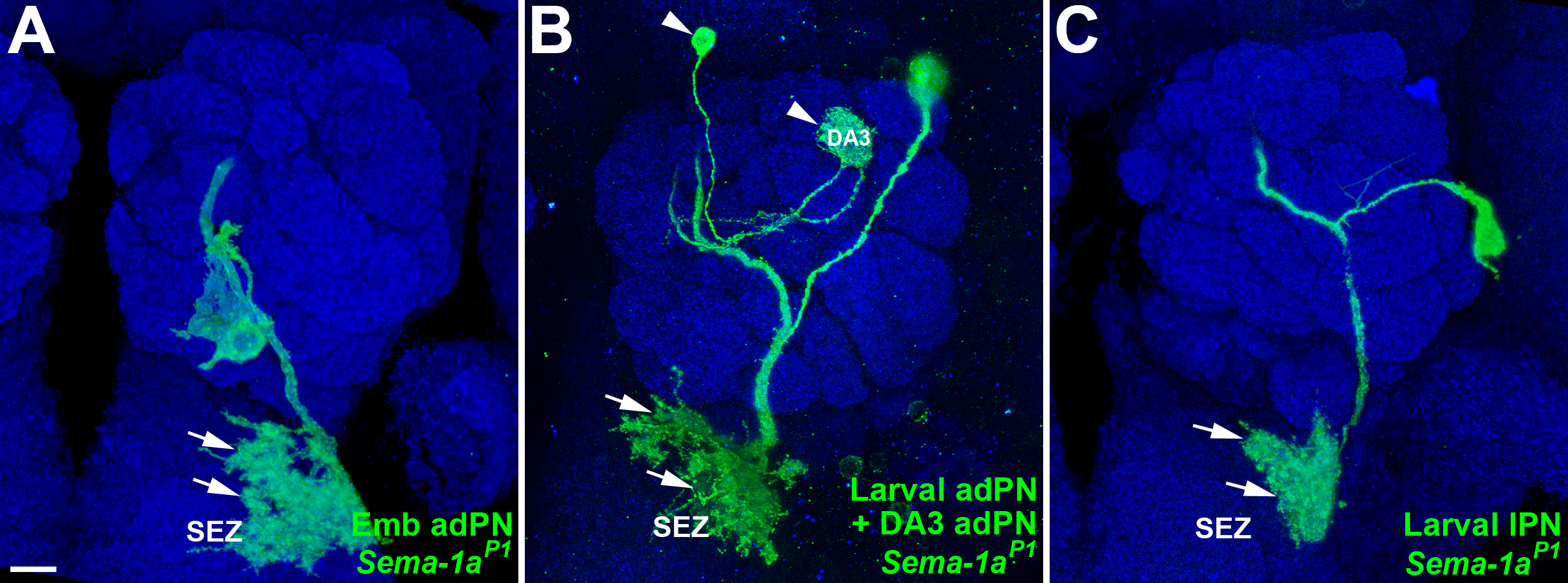

Supplement: S10 Fig — Confocal images of embryonic-born (Emb) adPNs and larval-born (Larval) adPNs and lPNs (green; labeled by GAL4-GH146) were used to reveal their dendritic morphologies in the AL and the SEZ. (A-C) Dendritic mis-targeting to the SEZ was found in many embryonic-born Sema-1aP1 adPNs and a few larval-born Sema-1aP1 adPNs and lPNs (arrows). A single Sema-1aP1 DA3 adPN was also observed in panel B (arrowheads). Brain neuropiles (shown in blue) were stained with antibody against Brp. Scale bar: 10 μm. (TIF) [file pgen.1006751.s010.tif]
