# Supplementary material for: Semaphorin-1a prevents Drosophila olfactory projection neuron dendrites from mis-targeting into select antennal lobe regions
Source: PLoS Genet. 2017 Apr 27;13(4):e1006751. doi: 10.1371/journal.pgen.1006751 (PMC5426794; doi:10.1371/journal.pgen.1006751)
Supplement: S1 Table — (PDF) [file pgen.1006751.s011.pdf]

S1 Table: Phenotypic description of PNs in *wild-type*, *Sema-1a LOF* and rescue experiments in S4 and S6 Figures

	type of PN (emb/larval)[a]	conditions of Sema-1a expression	total number (n)	% of obvious dendritic mis-targeting	% of dendritic occupancy in Brp negative regions and/or partial glomerulus	% of other dendritic mis-targeting	notes
Dendrites of *Sema-1a*-deficient PNs do not mis-target into select AL regions	VA2 adPN (emb)	*wt*	1	0%	0%	0%	
		Sema-1a^{P1}	3	0%	0%	0%	
	VM3 adPN (emb)	*wt*	6	0%	0%	0%	
		Sema-1a^{P1}	9	11%[b]	0%	22%[d]	[b] dendritic splitting (11%)<br>[d] mis-targeting of a few dendritic arbors to the DM1 (11%) and DM5 (11%) glomeruli
	VA3 adPN (larvaln)	*wt*	2	0%	0%	0%	
		Sema-1a^{P1}	5	40%[b]	0%	0%	[b] dendritic innervation in the ventral edge of the VM5v glomerulus
		Sema-1aP1+Sema-1a	5	0%	80%[c]	0%	[c] dendritic innervation in the ventral part and/or largely outside of the DL1 glomerulus (Brp negative region)
	DL3 lPN (larval; S6 Fig.)	*wt*	13	0%[b]	0%	0%	
		Sema-1a^{P1}	14	0%	0%	0%	
		Sema-1aP1+Sema-1a	18	0%	100%[c]	11%[d]	[c] dendritic innervation outside of the DL3 glomerulus (Brp negative region)<br>[d] dendritic splitting
	VA4 lPN (larval)	*wt*	2	0%	0%	0%	
		Sema-1a^{P1}	2	50%[b]	0%	0%	[b] dendritic splitting
	VC1 lPN (larval)	*wt*	1	0%	0%	0%	
		Sema-1a^{P1}	17	6%[b]	0%	0%	[b] dendritic innervation to the region ventral to the DP1m glomerulus
	VC2 lPN (larval)	*wt*	2	0%	0%	0%	
		Sema-1a^{P1}	2	0%	0%	0%	
	VA1lm vPN (larval; S4 Fig.)	*wt*	5	0%	0%	0%	
		Sema-1a^{P1}	15	0%	0%	0%	
		Sema-1aP1+Sema-1a	8	0%	0%	0%	
	VL1 vPN (larval; S4 Fig.)	*wt*	4	0%	0%	0%	
		Sema-1a^{P1}	11	0%	0%	0%	
		Sema-1aP1+Sema-1a	3	0%	0%	0%	

[a] emb:embryonic-born; larval: larval-born

[b, c, d] see the description in the column of notes
