# Supplementary material for: Semaphorin-1a prevents Drosophila olfactory projection neuron dendrites from mis-targeting into select antennal lobe regions
Source: PLoS Genet. 2017 Apr 27;13(4):e1006751. doi: 10.1371/journal.pgen.1006751 (PMC5426794; doi:10.1371/journal.pgen.1006751)
Supplement: S2 Table — (PDF) [file pgen.1006751.s012.pdf]

S2 Table. Generation of specific types of *Sema-1a*^{P1} adPNs in the synchronized MARCM experiment based on their birth-order

genotype: *hs-FLP*^{122}*/+;FRT*^{40A}*,UAS-mCD8::GFP,Sema1a*^{P1}*,GAL4-GH146/FRT*^{40A}*,tub-GAL80;+;+*

adPN \ hr ALH	30-34 (n=15)	34-38 (n=26)	38-42 (n=9)	42-46 (n=10)	46-50 (n=8)
DL1	100%	19%	0%	0%	0%
DA3	0%	38%	0%	9%	10%
DC2	0%	27%	44%	27%	0%
D	0%	8%	56%	18%	10%
VA3	0%	0%	0%	18%	30%
DC3	0%	8%	0%	19%	30%
VA1d	0%	0%	0%	9%	20%
