# Supplementary material for: Semaphorin-1a prevents Drosophila olfactory projection neuron dendrites from mis-targeting into select antennal lobe regions
Source: PLoS Genet. 2017 Apr 27;13(4):e1006751. doi: 10.1371/journal.pgen.1006751 (PMC5426794; doi:10.1371/journal.pgen.1006751)
Supplement: S3 Table — (PDF) [file pgen.1006751.s013.pdf]

S3 Table. Generation of specific types of adPNs in the *Sema-1a*^{P1} mutant with ectopic *Sema-1a* expression in the synchronized MARCM experiment based on their birth-order

genotype: *hs-FLP*^{122}*/+;FRT*^{40A}*,UAS-mCD8::GFP,Sema1a*^{P1}*,GAL4-GH146/FRT*^{40A}*,tub-GAL80;UAS-Sema-1a*^{sy}*/+;+*

adPN \ hr ALH	26-30 (n=5)	30-34 (n=24)	34-38 (n=3)	38-42 (n=16)	42-46 (n=15)	46-50 (n=15)	50-54 (n=4)
DL1	60%	33%	0%	0%	0%	0%	0%
DA3(DL3/DA4l)	20%	38%	0%	6%	7%	0%	0%
DC2	0%	8%	67%	6%	0%	0%	0%
D	20%	21%	0%	31%	0%	13%	0%
VA3	0%	0%	0%	13%	13%	7%	0%
DC3	0%	0%	33%	25%	40%	60%	50%
VA1d	0%	0%	0%	19%	40%	20%	50%
