# Supplementary material for: Semaphorin-1a prevents Drosophila olfactory projection neuron dendrites from mis-targeting into select antennal lobe regions
Source: PLoS Genet. 2017 Apr 27;13(4):e1006751. doi: 10.1371/journal.pgen.1006751 (PMC5426794; doi:10.1371/journal.pgen.1006751)
Supplement: S4 Table — (PDF) [file pgen.1006751.s014.pdf]

S4 Table: Phenotypic description of PNs in *wild-type*, *Sema-1a LOF* and rescue experiments in S8 and S9 Figures

	type of PN (emb/larval)[a]	conditions of Sema-1a expression	total number (n)	% of dendritic mis-targeting in the VM7v glomerulus	% of dendritic occupancy in Brp negative regions and/or partial glomerulus	% of other dendritic mis-targeting	notes
Sema-1a-deficient PNs with a phenotype of dendritic mis-targeting to the VM7v glomerulus	DM1 lPN[e] (larval; S9 Fig.)	*wt*	6	0%	0%	0%	
		Sema-1a^{P1}	6	83%[b]	0%	50%[d]	[b] dendritic innervation in the VM7v glomerulus<br>[d] dendritic innervation in the VA4 glomerulus (33%; one: heavy dendritic innervation; the other one: a dendritic branch) and the region around the VC1 glomerulus (17%)
		Sema-1a^{P1} +Sema-1a	7	0%	14%[c]	14%[d]	[c] dendritic innervation largely outside of the DM1m glomerulus (Brp negative region)<br>[d] a patch of dendritic innervation ventral to the VM7v glomerulus
	DM2 lPN (larval; S9 Fig.)	*wt*	8	0%	0%	0%	
		Sema-1a^{P1}	26	31%[b]	0%	27%[d]	[b] dendritic innervation in the VM7v glomerulus<br>[d] dendritic innervation to the region posterior to the VM7d glomerulus (12%) and the region ventral to the DP1m, DM1 or DM2 glomerulus (19%)
		Sema-1a^{P1} +Sema-1a	8	0%	100%[c]	0%	[c] dendritic innervation in the dorsal (50%) or ventral (50%) part of the DM2 glomerulus

	type of PN (emb/larval)[a]	conditions of Sema-1a expression	total number (n)	% of dendritic mis-targeting in the VA4 glomerulus	% of dendritic occupancy in Brp negative regions and/or partial glomerulus	% of other dendritic mis-targeting	notes
Sema-1a-deficient PNs with a phenotype of dendritic mis-targeting to the VA4 glomerulus	DM1 lPN[e] (larval; S9 Fig.)	*wt*	6	0%	0%	0%	
		Sema-1a^{P1}	6	50%[b]	0%	83%[d]	[b] dendritic innervation in the VA4 glomerulus (33%; one: heavy dendritic innervation; the other one: a dendritic branch) and the region around the VC1 glomerulus (17%)<br>[d] dendritic innervation in the VM7v glomerulus
		Sema-1a^{P1} +Sema-1a	7	0%	14%[c]	14%[d]	[c] dendritic innervation largely outside of the DM1m glomerulus (Brp negative region)<br>[d] a patch of dendritic innervation ventral to the VM7v glomerulus
	VA7m lPN[e] (larval; S9 Fig.)	*wt*	4	25%[b]	0%	0%	[b] a small patch of dendritic innervation to the VA4 glomerulus
		Sema-1a^{P1}	9	67%[b]	0%	44%[d]	[b] dendritic innervation in part of the VA4 and/or VC2 glomeruli<br>[d] dendritic innervation to the region close to the VC1 glomerulus
		Sema-1a^{P1} +Sema-1a	15	0%	100%[c]	0%	[c] dendritic innervation largely outside of the VA7m glomerulus (Brp negative region)

	type of PN (emb/larval)[a]	conditions of Sema-1a expression	totoal number (n)	% of dendritic mis-targeting in the DP1m glomerulus	% of dendritic occupancy in Brp negative regions and/or partial glomerulus	% of other dendritic mis-targeting	notes
Sema-1a-deficient PNs with a phenotype of dendritic mis-targeting to the region ventral to the DP1m glomerulus	DL1 adPN (larval; S8 Fig.)	*wt*	12	0%	0%	0%	
		Sema-1a^{P1}	35	17%[b]	0%	94%[d]	[b] dendritic innervation to the region ventral to the DP1m glomerulus<br>[d] dendritic mis-targeting along the DL-to-VM axis of the AL as described previously (Komiyama *et al.*, 2007)
		Sema-1a^{P1} +Sema-1a	12	0%	100%[c]	0%	[c] dendritic innervation largely outside of the DL1 glomerulus (Brp negative region)
	DL5 lPN (emb; S9 Fig.)	*wt*	6	0%	0%	17%[d]	[d] a patch of dendritic arbors anteroventral to the DP1m glomerulus
		Sema-1a^{P1}	4	50%[b]	0%	25%[d]	[b] dendritic innervation to the region ventral to the DP1m glomerulus<br>[d] dendritic splitting
	DM3 adPN (emb; S9 Fig.)	*wt*	3	0%	0%	0%	
		Sema-1a^{P1}	4	25%[b]	0%	25%[d]	[b] dendritic innervation to the region ventral to the DP1m glomerulus<br>[d] a patch of dendritic innervation posterior to the VC1 glomerulus

[a] emb:embryonic-born; larval: larval-born

[b, c, d] see the description in the column of notes

[e] same types of PNs can be also seen in different classes of dendritic mis-targeting phenotypes in Table 2 and this supporting table
