# Supplementary material for: Semaphorin-1a prevents Drosophila olfactory projection neuron dendrites from mis-targeting into select antennal lobe regions
Source: PLoS Genet. 2017 Apr 27;13(4):e1006751. doi: 10.1371/journal.pgen.1006751 (PMC5426794; doi:10.1371/journal.pgen.1006751)
Supplement: S5 Table — (PDF) [file pgen.1006751.s015.pdf]

S5 Table: Phenotypic description of PNs in *wild-type*, *Sema-1a LOF* and rescue experiments in Figures 1 and 4 and S5, S6 and S10 Figures

	type of PN (emb/larval)[a]	conditions of Sema-1a expression	total number (n)	% of dendritic mis-targeting to the SEZ	% of dendritic occupancy in Brp negative regions and/or partial glomerulus	% of other dendritic mis-targeting	notes
Sema-1a-deficient PNs with a phenotype of dendritic mis-targeting to the SEZ	DA4m adPN[e] (emb; S5 Fig.)	*wt*	4	0%	0%	25%[d]	[d] a few dendritic arbors around the DC1 glomerulus
		Sema-1a^{P1}	4	100%[b]	0%	50%[d]	[b] dendritic innervation in the SEZ<br>[d] partial dendritic innervation in the DA3 glomerulus
	VL2p adPN[e] (emb; Fig.4)	*wt*	43	0%	0%	14%[d]	[d] some dendritic arbors posterior to DP1m, V or VL1 glomerulus or posterior to the AL (9%) and b a patch of dendritic arbors ventrally around the VC1 glomerulus (5%)
		Sema-1a^{P1}	15	87%[b]	0%	87%[d]	[b] dendritic innervation to the SEZ (13% out of 87% without the innervation around the VC1 glomerulus)<br>[d] dendritic innervation mostly to the region posterior to the VC1 glomerulus (13% out of 87% without the SEZ innervation)
	DA1 lPN (larval; S6 Fig.)	*wt*	37	0%	45%[c]	0%	[c] dendritic innervation in the ventral (29%) or dorsal (16%) parts of the DA1 glomerulus
		Sema-1a^{P1}	27	19%[b]	89%[c]	26%[d]	[b] dendritic innervation to the region ventral to the DP1m glomerulus<br>[c] dendritic innervation in the dorsal part of the DA1 glomerulus (89%)<br>[d] dendritic mis-targeting along the DL-to-VM axis of the AL as described previously (Komiyama *et al.*, 2007)
		Sema-1a^{P1} +Sema-1a	24	0%	100%[c]	0%	[c] dendritic innervation in the ventral part and/or outside of the DA1 glomerulus (Brp negative region)
	diffuse vPN[e] (larval; Fig.1)	*wt*	6	0%	0%	0%	
		Sema-1a^{P1}	4	100%[b]	0%	100%[d]	[b] dendritic innervation in the SEZ<br>[d] heavy dendritic accumulation in the DA3 glomerulus
		Sema-1aP1+Sema-1a	7	0%	100%[c]	29%[d]	[c] no/rarely dendritic innervation in the DA3 glomerulus<br>[d] dendritic innervation across midline to the contralateral AL
	SEZ adPN (emb; S10 Fig.)	*Sema-1a*^{P1}	17	100%[b]	0%	0%	[b] dendritic innervation to the SEZ and no dendritic innervation in the AL
	SEZ adPN (larval; S10 Fig.)	*Sema-1a*^{P1}	3	100%[b]	0%	0%	[b] dendritic innervation to the SEZ and no dendritic innervation in the AL
	SEZ lPN (larval; S10 Fig.)	*Sema-1a*^{P1}	3	100%[b]	0%	0%	[b] dendritic innervation to the SEZ and no dendritic innervation in the AL

[a] emb:embryonic-born; larval: larval-born
[b, c, d] see the description in the column of notes
[e] same types of PNs can be also seen in different classes of dendritic mis-targeting phenotypes in Tables 1 and 2
