# Supplementary material for: Semaphorin-1a prevents Drosophila olfactory projection neuron dendrites from mis-targeting into select antennal lobe regions
Source: PLoS Genet. 2017 Apr 27;13(4):e1006751. doi: 10.1371/journal.pgen.1006751 (PMC5426794; doi:10.1371/journal.pgen.1006751)
Supplement: S6 Table — (PDF) [file pgen.1006751.s016.pdf]

S6 Table. Genotypes of the flies in the figure panels.

Figure	Genotype
S1A,S1B,S2A-S2H	*hs-FLP*^{122}*/w;UAS-mCD8::GFP,UAS-rCD2RNAi,FRT*^{40A}*,GAL4-GH146/ UAS-rCD2::RFP,UAS-GFPRNAi,FRT*^{40A}*;GAL4-MZ699/+;+*
1A,1D,2B,2E,2H,2K,2O,2Q,3A, 4A,4C,4E,4G,4I,S4A,S4D,S6A-S6C,S6G,S8A,S9A,S9C,S9E,S9G, S9I,S9K	*hs-FLP*^{122}*/w;UAS-mCD8::GFP,FRT*^{40A}*,GAL4-GH146/tubP-GAL80,FRT*^{40A}*;+;+*
1B,1E,2C,2F,2I,2L,2P,2R,3B,4B,4D,4F,4H,4J-4L,S4B,S4E,S6D, S6E,S6H,S8B,S9B,S9D,S9F,S9H, S9J,S9L,S10A-S10C	*hs-FLP*^{122}*/w;UAS-mCD8::GFP,Sema-1a*^{P1}*,FRT*^{40A}*,GAL4-GH146/tubP-GAL80, FRT*^{40A}*;+; +*
1C,1F,2D,2G,2J,3C-3F,S4C,S4F, S6F,S6I,S7A-S7C,S8C	*hs-FLP*^{122}*/w;UAS-mCD8::GFP,Sema-1a*^{P1}*,FRT*^{40A}*,GAL4-GH146/tubP-GAL80, FRT*^{40A}*; UAS-Sema-1a*^{sy}*/+;+*
S1C	*hs-FLP*^{122}*/w;UAS-mCD8::GFP,UAS-rCD2RNAi,FRT*^{40A}*/tubP-GAL80,FRT*^{40A}*; actin-FRT<stop<FRT-GAL4/+;+*
2M&S5A	*hs-FLP*^{122}*/w;UAS-mCD8::GFP,UAS-rCD2RNAi,FRT*^{40A}*/tubP-GAL80,FRT*^{40A}*; R38B04-GAL4/+;+*
2N	*hs-FLP*^{122}*/w;UAS-mCD8::GFP,UAS-rCD2RNAi,FRT*^{40A}*/tubP-GAL80,FRT*^{40A}*; R38B04-GAL4/UAS-Sema-1aRNAi*^{TRiP}*;+*
S3A	*w;GH146-FLP/+;R95B09-GAL4,UAS-FRT<stop<FRT-myr-GFP/+;+*
S3B-S3D	*w;GH146-FLP/+;R95B09-GAL4,UAS-FRT<stop<FRT-myr-GFP/ UAS-Sema-1aRNAi*^{TRiP}*;+*
S3E	*hs-FLP*^{122}*/w;UAS-mCD8::GFP,UAS-rCD2RNAi,FRT*^{40A}*/tubP-GAL80,FRT*^{40A}*; R95B09-GAL4/UAS-Sema-1aRNAi*^{TRiP}*;+*
S5B-S5D	*hs-FLP*^{122}*/w;UAS-mCD8::GFP, Sema-1a*^{P1}*,FRT*^{40A}*,GAL4-MZ19/ tubP-GAL80,FRT*^{40A}*;R38B04-GAL4/+;+*
